# CircSMAD3 represses VSMC phenotype switching and neointima formation via promoting hnRNPA1 ubiquitination degradation

**DOI:** 10.1111/cpr.13742

**Published:** 2024-09-01

**Authors:** Shuai Mei, Xiaozhu Ma, Li Zhou, Qidamugai Wuyun, Ziyang Cai, Jiangtao Yan, Hu Ding

**Affiliations:** ^1^ Division of Cardiology, Department of Internal Medicine, Tongji Hospital, Tongji Medical College Huazhong University of Science and Technology Wuhan China; ^2^ Hubei Key Laboratory of Genetics and Molecular Mechanisms of Cardiological Disorders Wuhan China

## Abstract

Circular RNAs (circRNAs) are novel regulatory RNAs with high evolutionary conservation and stability, which makes them effective therapeutic agents for various vascular diseases. The SMAD family is a downstream mediator of the canonical transforming growth factor beta (TGF‐β) signalling pathway and has been considered as a critical regulator in vascular injury. However, the role of circRNAs derived from the SMAD family members in vascular physiology remains unclear. In this study, we initially identified potential functional circRNAs originating from the SMAD family using integrated transcriptome screening. circSMAD3, derived from the SMAD3 gene, was identified to be significantly downregulated in vascular injury and atherosclerosis. Transcriptome analysis was conducted to comprehensively illustrate the pathways modulated by circRNAs. Functionally, circSMAD3 repressed vascular smooth muscle cell (VSMC) proliferation and phenotype switching in vitro evidenced by morphological assays, and ameliorated arterial injury‐induced neointima formation in vivo. Mechanistically, circSMAD3 interacted with heterogeneous nuclear ribonucleoprotein A1 (hnRNPA1) within the nucleus, enhanced its interaction with E3 ligase WD repeat domain 76 to promote hnRNPA1 ubiquitination degradation, facilitated p53 pre‐RNA splicing, activated the p53γ signalling pathway, and finally suppressed VSMC proliferation and phenotype switching. Our study identifies circSMAD3 as a novel epigenetic regulator that suppresses VSMC proliferation and phenotype switching, thereby attenuating vascular remodelling and providing a new circRNA‐based therapeutic strategy for cardiovascular diseases.

## Graphical abstract

Briefly, circSMAD3 recruits hnRNPA1 to enhance the interaction of hnRNPA1 with WDR76, promote its ubiquitination degradation, facilitate p53 pre‐RNA splicing to activate the p53γ signalling pathway, and eventually maintain the vascular homeostasis. In vascular injury, the downregulated circSMAD3 promotes hnRNPA1 function, deactivates p53γ signalling pathway, and leads to neointima formation.

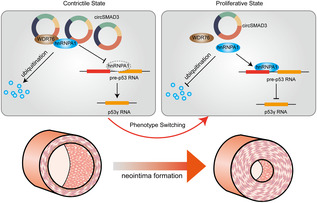

## 1 INTRODUCTION

Vascular smooth muscle cells (VSMCs) are the prominent cells in the blood vessel wall that can transition from a contractile state to a synthetic state in a process known as ‘phenotype switching’,^1^, ^2^ followed by the reduction of differentiation markers,^3^, ^4^ such as alpha‐smooth muscle actin (α‐SMA), transgelin, and calponin. This VSMC characteristic leads to arterial lumen stenosis or structural abnormalities when an arterial injury occurs, which triggers the occurrence of vascular proliferative diseases, including atherosclerosis and restenosis after angioplasty, and eventually increases the risk of myocardial infarction.^5^ However, the underlying mechanisms of VSMC phenotype switching remain unclear.

As a novel type of non‐coding RNA, circular RNAs (circRNAs) significantly affect the progression, diagnosis, and treatment of human diseases, including cardiovascular diseases.^6^, ^7^, ^8^, ^9^, ^10^ They act as microRNA sponges,^11^ protein modulators,^12^, ^13^ genetic transcription regulators,^14^ and translation templates^15^, ^16^ in pathophysiological processes, including circ_Lrp6 and circEsyt2 in vascular proliferative diseases.^17^, ^18^ circRNAs are considered more stable and have recently attracted more attention for the recent development of synthetic circRNAs as theranostics and vaccines due to their covalently closed‐loop structures and low immunity.^19^, ^20^, ^21^, ^22^ Notably, some attempts have been made to develop circRNA‐based therapies, and great progress has been made. For example, circRNAs have been successfully studied as vaccines for overcoming the coronavirus disease 2019 pandemic by engineering circRNAs that encode severe acute respiratory syndrome coronavirus 2 receptor‐binding domain.^23^, ^24^ These studies have revealed the great value and broad application prospects of circRNAs in cardiovascular diseases.^25^, ^26^ Specifically, these results have shown that circRNAs have great potential as novel therapies for addressing cardiovascular diseases.

The SMAD family, as mediators of the canonical transforming growth factor‐beta (TGF‐β) signalling pathway, reportedly plays a critical role in vascular homeostasis.^27^, ^28^, ^29^ Notably, several studies have reported that mutations in the SMAD family members lead to angiodysplasia or aortic aneurysm and dissection syndromes.^30^, ^31^, ^32^ However, the role of circRNAs derived from the SMAD family members in vascular physiology remains unclear. Therefore, in this study, we aimed to identify circSMAD3 to fully illustrate its vital functions in VSMC phenotype switching and proliferation in vitro and in vivo by enhancing the interaction of heterogeneous nuclear ribonucleoprotein A1 (hnRNPA1) with E3 ligase WD repeat domain 76 (WDR76), repressing the splicing of p53 precursor RNA (p53 pre‐RNA), and facilitating the p53γ signalling pathway. Our study demonstrates the critical role of circSMAD3 in patients with vascular injury and provides novel targets for developing circRNA‐based therapies for cardiovascular diseases.

## 2 RESULTS

### 2.1 CircSMAD3 is a conserved circRNA downregulated in response to vascular injury

**FIGURE 1:**
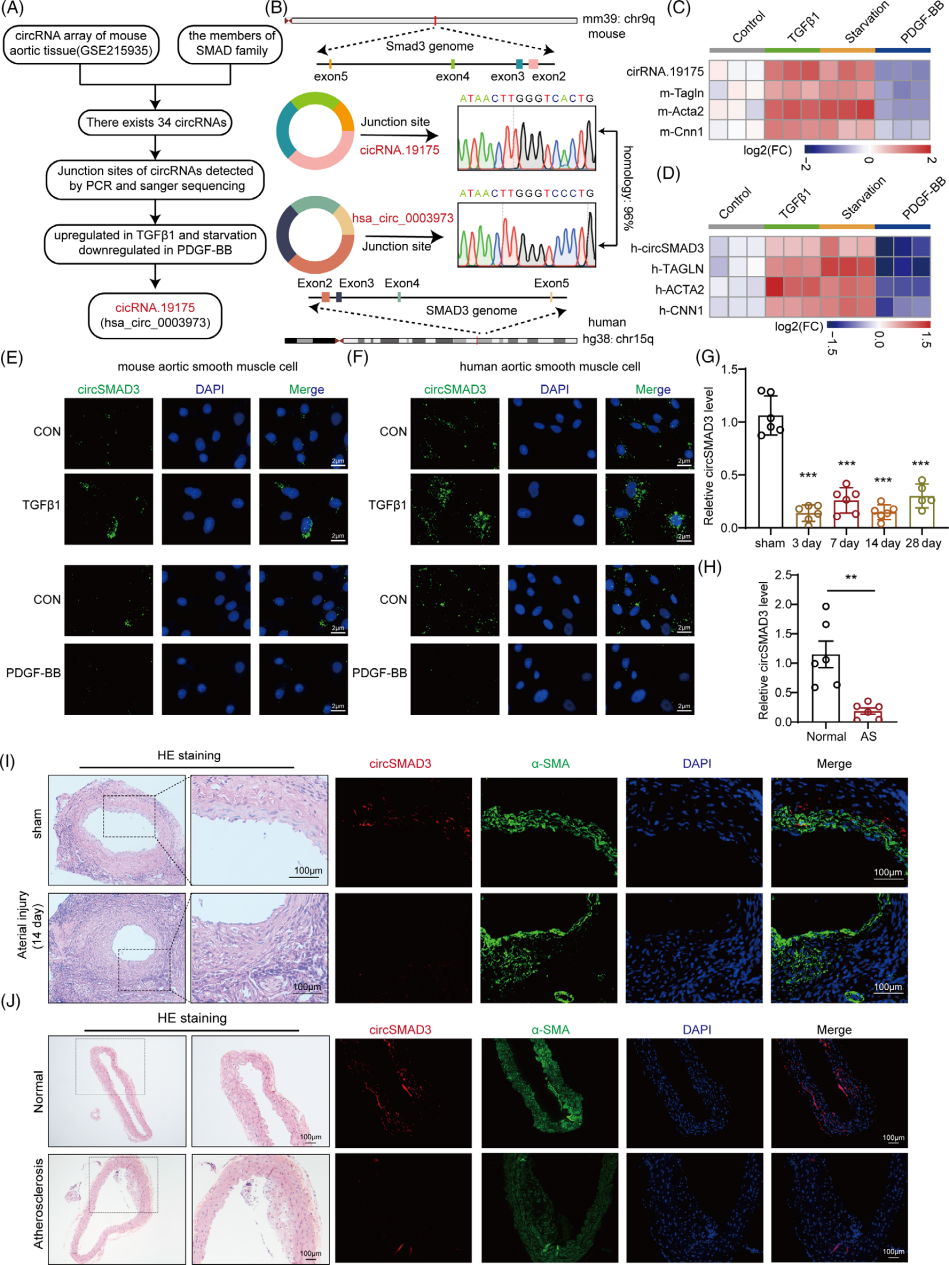
circSMAD3 was significantly downregulated in response to vascular diseases. (A) Schematic identification of circRNAs derived from the members of the SMAD family. (B) Schematic of circSMAD3 derived from SMAD3 gene in mice and humans, and their junction sites detected using the Sanger sequencing. (C, D) Heat map showing the changes in circSMAD3 in response to TGF‐β1 (10 ng/mL), PDGF‐BB (20 ng/mL), and starvation for 24 h in mASMCs (C) and HASMCs (D). Data are shown as mean ± SD (one‐way ANOVA) (E, F) Fluorescence in situ hybridization (FISH) showing the detection of circSMAD3 expression in response to TGF‐β1 (10 ng/mL), PDGF‐BB (20 ng/mL), and starvation for 24 h in mASMCs (E) and HASMCs (F). Scale bar = 2 μm. (G) circSMAD3 expression in the time points of arterial injury detected using qPCR. *N* = 6. Data are shown as mean ± SEM (one‐way ANOVA) (H) The level of circSMAD3 in atherosclerotic plaque compared with normal aortic tissues using qPCR (*N* = 6). Data are shown as mean ± SEM (Student's t‐test) (I) FISH showing the detection of circSMAD3 changes in the injured arterial tissue at 14 days. Scale bar = 100 μm. (J) FISH showing the detection of circSMAD3 changes in atherosclerotic plaque. Scale bar = 100 μm. ***p<0.001, **p<0.01.

We initially performed genomic screening by integrating the circBase database and circRNA microarray data (GSE215935) from mouse aortic tissue to identify the critical vascular remodelling‐related circRNAs. We also focused on this gene family to search for functional circRNAs since the SMAD family plays a central role in regulating TGF‐β1 and is involved in vascular injury.^33^ Overall, 34 circRNAs from SMAD1 to SMAD9 genes were identified (Figure 1A and Figure S1A). Among them, a previously uncharacterized circRNA, circRNA.19175, which was derived from two to five exons of the SMAD3 gene, known as circSMAD3 (Figure 1B), was upregulated in response to the stimulation of TGF‐β1 or serum deprivation and downregulated in response to platelet‐derived growth factor subunit B (PDGF‐BB) in mouse primary aortic smooth muscle cells (mASMCs) (Figure 1C,E and Figure S1B,C). This observation was notable. A conserved human circRNA, hsa_circ_0003973, which contains a homologous junction site for circSMAD3, was also detected in human aortic smooth muscle cells (HASMCs) (Figure 1B). Consistent with the findings of mASMCs, hsa_circ_0003973 was largely altered in HASMCs after stimulation using TGF‐β1, PDGF‐BB, and starvation (Figure 1D,F).

We also explored the properties of circSMAD3 in HASMCs and observed that circSMAD3 was more stable than linear RNA in response to actinomycin D stimulation at different times or RNase R intervention (Figure S1E,F). Furthermore, circSMAD3 is only detected by random primer rather than the oligo primer, as no poly(A) tail was observed (Figure S1G). These results demonstrated that circSMAD3 is a bona fide circRNA in HASMCs.

In the vascular injury model, circSMAD3 gradually declined in parallel in the carotid artery on days 3, 7, 14, and 28 (Figure 1G,I). Furthermore, circSMAD3 levels were downregulated in mouse atherosclerotic plaques (Figure 1H,J). Therefore, these results indicate that circSMAD3 is involved in the pathogenesis of vascular proliferative diseases.

### 2.2 CircSMAD3 inhibits vascular smooth muscle cell phenotype switching and proliferation in vitro

Furthermore, we explored the function of circSMAD3 in phenotype switching and proliferation in vitro by infecting mASMCs with sh‐circSMAD3 and circSMAD3 overexpressing adenoviruses, respectively. CircSMAD3 was silenced and overexpressed by the corresponding virus, and the parent gene had no influence (Figure S2A). CircSMAD3 silencing significantly promoted phenotype switching by downregulating contractile marker genes, such as α‐SMA, smooth muscle protein 22 alpha (SM22α), and Calponin 1 (CNN1), whereas circSMAD3 overexpression dramatically increased these gene expressions (Figure S2A,B). Furthermore, circSMAD3 silencing resulted in the switching of VSMCs from elongated contractile morphology to a polygonal synthetic morphology and reversed its contraction effect induced by TGF‐β1; however, circSMAD3 overexpression maintained the contractile state of mASMCs (Figure S2D,E). Therefore, we performed Transwell, wound healing, 5‐Ethynyl‐2′‐deoxyuridine (EdU), and cell counting kit‐8 assays to further investigate the ability of circSMAD3 to affect VSMC proliferation. circSMAD3 silencing substantially promoted the proliferation and migration capacities of VSMCs and receded the effects induced by TGF‐β1, whereas its overexpression suppressed these effects (Figure S2C,F–H).

**FIGURE 2:**
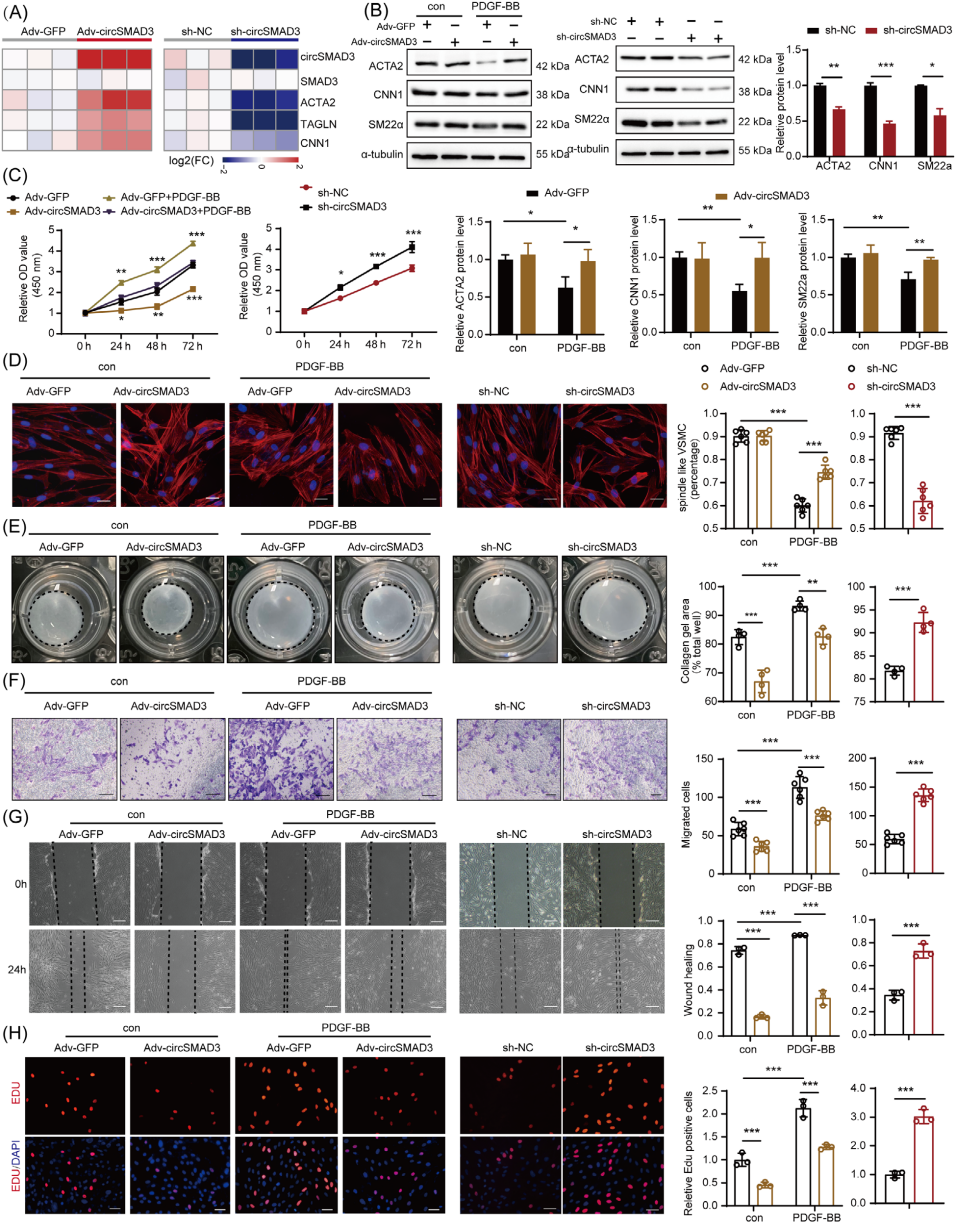
circSMAD3 modulates VSMC phenotype switching and proliferation in HASMCs. (A) Heat map showing the overexpression and silencing efficiency after being infected with circSMAD3 overexpressing or silencing adenovirus and the expression of contractile marker genes. Data are shown as mean ± standard deviation (SD) (Student's *t*‐test, as indicated). (B) Western blot showing the detection of the protein level expression of contractile genes with circSMAD3 overexpression and PDGF‐BB (20 ng/mL) (left) and silencing (right). Data are shown as mean ± SD (two‐way ANOVA). (C) CCK‐8 assay showing the detection of HASMC proliferation after circSMAD3 overexpression and silencing at multiple time points (0, 24, 48, and 72 h). Data are shown as mean ± SD (left, two‐way ANOVA; right, Student's *t*‐test). (D) Left, phalloidin staining showing the morphology of HASMC after circSMAD3 overexpression and silencing. Scale bar = 50 μm. Right, the percentages of spindle‐like (contractile phenotype) VSMC in each group. Data are shown as mean ± SD (left, two‐way ANOVA; right, Student's *t*‐test). (E) Left, collagen gel contrition assay shows HASMC contraction evaluation after circSMAD3 overexpression and silencing. Right, the contraction rate is quantified in each group. Data are shown as mean ± SD (left, two‐way ANOVA; right, Student's *t*‐test). (F) Left, Transwell assay shows HASMC migration evaluation after circSMAD3 overexpression and silencing. Scale bar = 200 μm. Right, the migration rate is quantified in each group. Data are shown as mean ± SD (left, two‐way ANOVA; right, Student's *t*‐test). (G) Left, wound healing assay to evaluate the migration of HASMC after circSMAD3 overexpression and silencing. Scale bar = 100 μm. Right, the migration rate is quantified in each group. Data are shown as mean ± SD (left, two‐way ANOVA; right, Student's *t*‐test). (H) Left, EdU assay showing the detection of HASMC proliferation after circSMAD3 overexpression and silencing. Scale bar = 100 μm. Right, the EdU positive rate is quantified in each group. Data are shown as mean ± SD (left, two‐way ANOVA; right, Student's *t*‐test). ***p<0.001, **p<0.01, *p<0.05.

Furthermore, we verified the function of circSMAD3 on HASMCs. Similar to the results in mASMCs, circSMAD3 exerted consistent effects on HASMC phenotype switching and proliferation and reversed the effects of PDGF‐BB in HASMCs (Figure 2A–H).

### 2.3 CircSMAD3 attenuates post‐injury neointima formation in vivo

**FIGURE 3:**
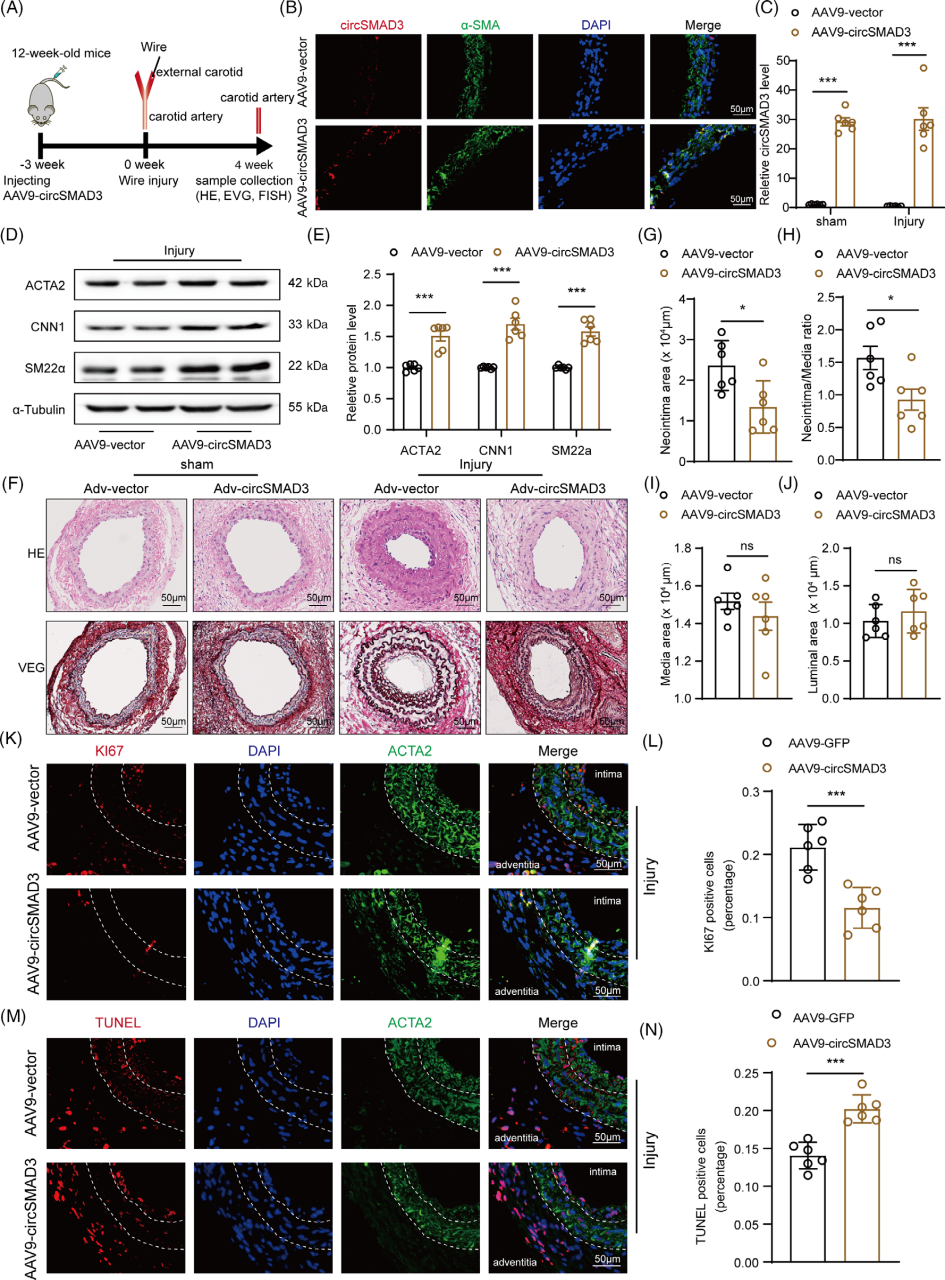
circSMAD3 ameliorated arterial injury‐induced neointima formation. (A) Schematic to show the construction of wire injury model accompanied by injection of circSMAD3‐overexpressing or control adeno‐associated virus with transgelin promoter in the carotid arteries from C57BL/6J mice. (B) Fluorescence in situ hybridization (FISH) and immunofluorescence showing the detection of circSMAD3 overexpression in aortic tissues. Scale bar = 50 μm. (C) qPCR assays showing the detection of the overexpression efficiency after being infected with circSMAD3 overexpressing adeno‐associated virus in carotid arterial tissues. Data are shown as mean ± SEM (two‐way ANOVA). *N* = 6. (D, E) Western blot showing the detection of the protein level expression of contractile genes with circSMAD3 overexpression using alpha‐tubulin as the internal reference. Data are shown as mean ± SEM (two‐way ANOVA). *N* = 6. (F) Haematoxylin and eosin staining showing the neointima formation caused by arterial injury and circSMAD3 overexpression. Scale bar = 50 μm. (G–J) Neointima area, Neointima/media ratio, media area, and luminal area in each group. Data are shown as mean ± SEM (Student's *t*‐test, as indicated). *N* = 6. (K‐L) Ki‐67 staining showing the VSMC proliferation caused by arterial injury and circSMAD3 overexpression (K). Scale bar = 50 μm. (L), the percentage of Ki‐67 positive cells in each group. Data are shown as mean ± SEM (Student's *t*‐test, as indicated). *N* = 6. (M, N) TUNEL staining showing the VSMC apoptosis caused by arterial injury and circSMAD3 overexpression (M). Scale bar = 50 μm. (N), the percentage of TUNEL‐positive cells in each group. Data are shown as mean ± SEM (Student's *t*‐test, as indicated). *N* = 6. ns, no significance, ***p<0.001, *p<0.05.

To assess the role of circSMAD3 in post‐injury neointima formation in vivo, C57BL/6J mice aged 12 weeks were infected with circSMAD3 overexpression adeno‐associated virus with a transgelin promoter. After 3 weeks, the right carotid artery was wire‐injured for another 4 weeks (Figure 3A). The efficiency of circSMAD3 overexpression was confirmed using Real‐time quantitative polymerase chain reaction (qPCR) and fluorescence in situ hybridization (Figure 3B,C). The expression of contractile genes, such as α‐SMA, SM22α, and CNN1, were significantly upregulated (Figure 3D,E). Additionally, histological analysis and quantification of the arterial neointima/media ratio showed that circSMAD3 overexpression repressed the wire injury‐induced neointima formation (Figure 3F–J). The percentage of Ki67‐positive cells representing the proliferation capacity decreased in the circSMAD3 overexpression group (Figure 3K,L). VSMC apoptosis also increased in vivo after circSMAD3 overexpression, as indicated by TUNEL staining (Figure 3M,N).

**FIGURE 4:**
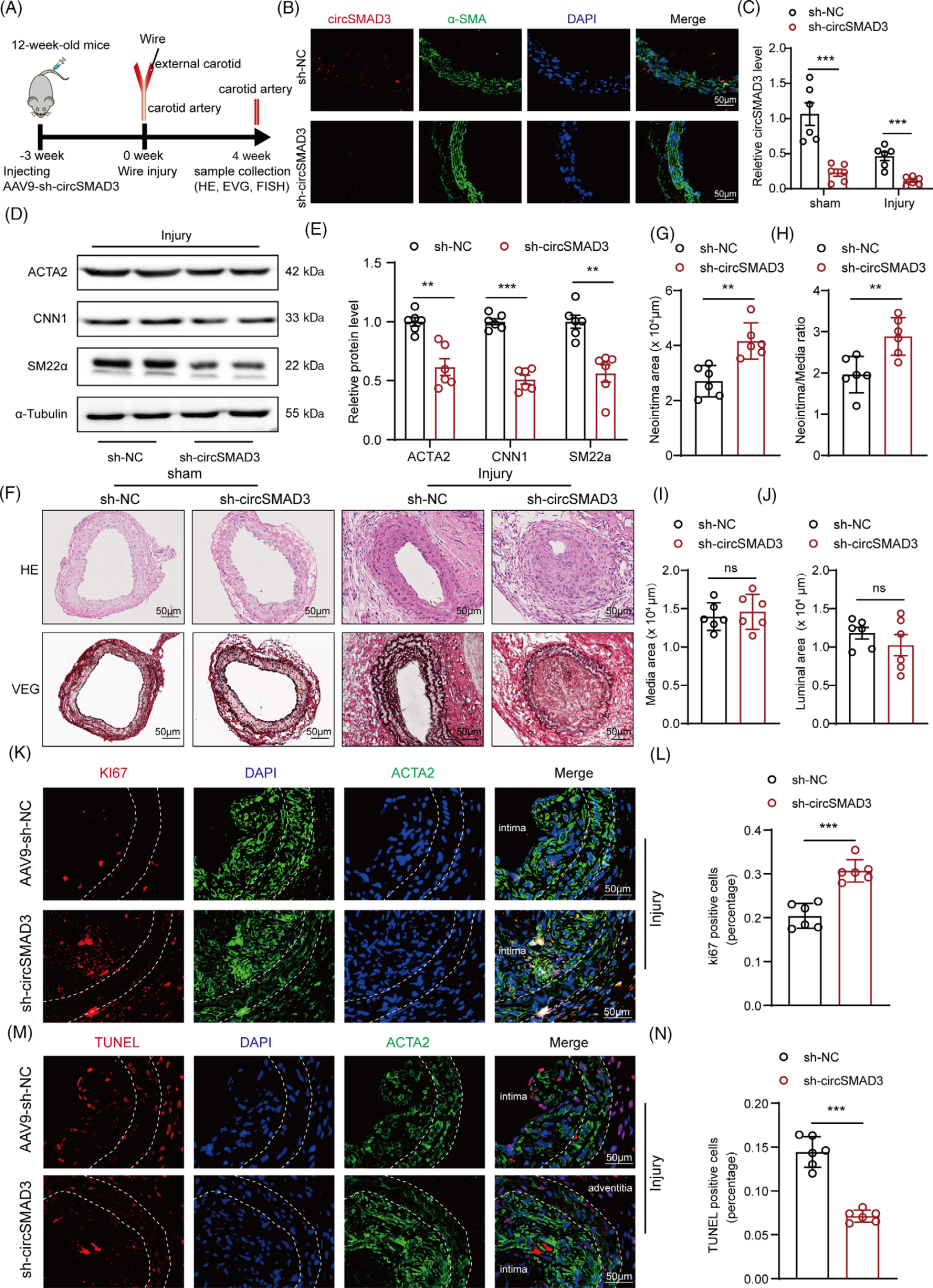
circSMAD3 knockdown aggravated arterial injury‐induced neointima formation. (A) Schematic to show the construction of wire injury model accompanied by injection of circSMAD3 short hairpin RNA or short hairpin‐negative control adeno‐associated virus with transgelin promoter in the carotid arteries from C57BL/6J mice. (B) Fluorescence in situ hybridization (FISH) and immunofluorescence showing the silence of circSMAD3 in aortic tissues. Scale bar = 50 μm. (C) qPCR assays showing the detection of the silencing efficiency after being infected with circSMAD3 knockdown adeno‐associated virus in carotid arterial tissues. Data are shown as mean ± SEM (two‐way ANOVA). *N* = 6. (D–E) Western blot showing the detection of the protein level expression of contractile genes with circSMAD3 silence in injured carotid arterial tissues using alpha‐tubulin as the internal reference. Data are shown as mean ± SEM (Student's *t*‐test). *N* = 6. (F) Haematoxylin and eosin staining showing the neointima formation caused by arterial injury and circSMAD3 silence. Scale bar = 50 μm. (G–J) Neointima area, Neointima/media ratio, media area, and luminal area in each group. Data are shown as mean ± SEM (Student's *t*‐test). *N* = 6. (K, L) Ki‐67 staining showing the VSMC proliferation caused by arterial injury and circSMAD3 silence (K). Scale bar = 50 μm. (L), the percentage of Ki‐67 positive cells in each group. Data are shown as mean ± SEM (Student's *t*‐test). *N* = 6. (M, N) TUNEL staining showing the VSMC apoptosis caused by arterial injury and circSMAD3 silence (M). Scale bar = 50 μm. (N), the percentage of TUNEL‐positive cells in each group. Data are shown as mean ± SD (Student's *t*‐test, as indicated). *N* = 6. ns, no significance, ***p<0.001, **p<0.01, *p<0.05.

We also performed the circSMAD3 knockdown experiment in vivo using the adeno‐associated virus system. Compared with the negative control, circSMAD3 silencing significantly promoted arterial injury‐induced neointima formation (Figure 4A–J). Consequently, circSMAD3 knockdown dramatically enhanced the injury‐induced proliferative capacity of VSMC and reduced the proportion of apoptotic cells (Figure 4K–N).

### 2.4 CircSMAD3 maintains VSMC constriction phenotype through the p53γ signalling pathway

**FIGURE 5:**
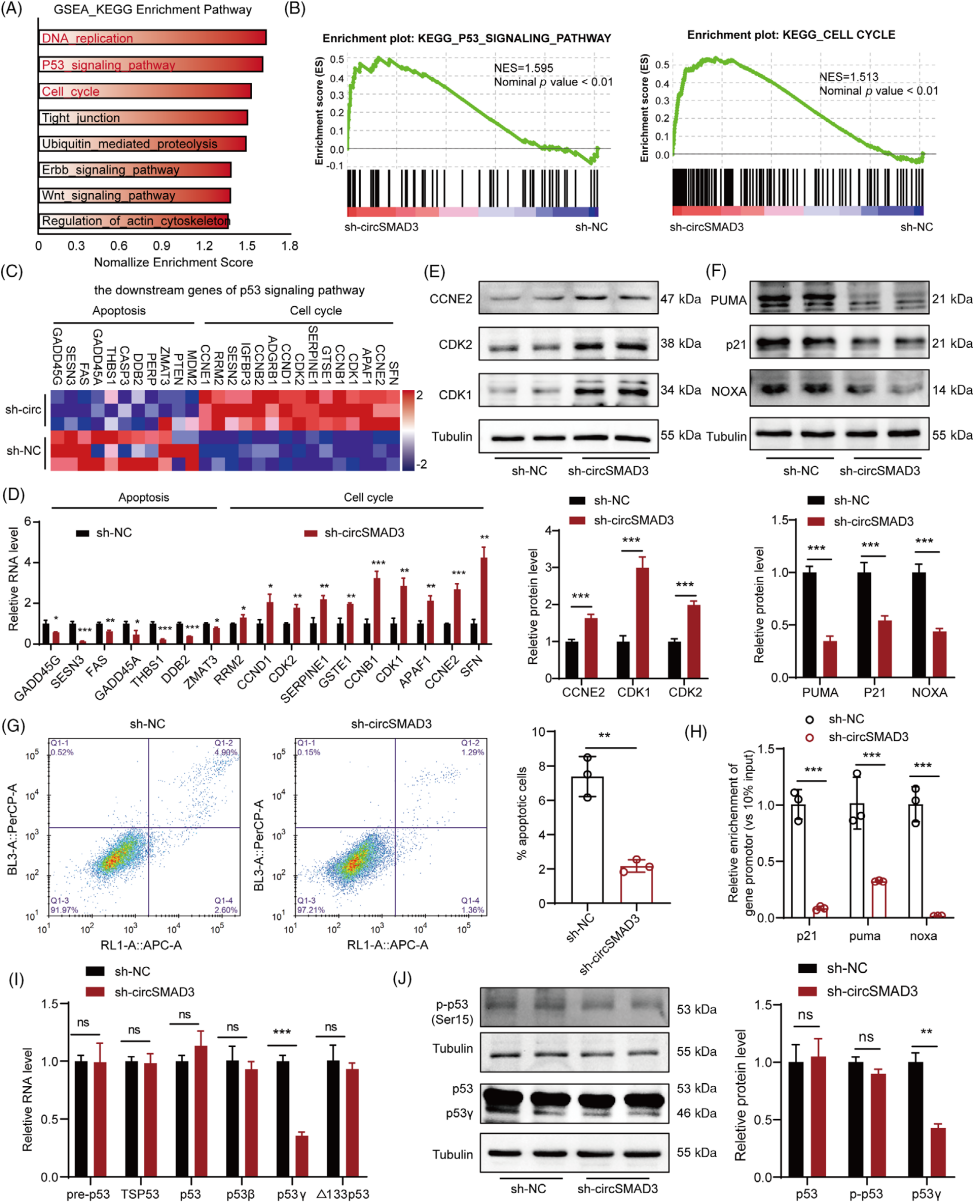
circSMAD3 maintains VSMC phenotype switching through the p53γ signalling pathway. (A) The pathways enriched by genes modulated by circSMAD3 silencing were analysed using gene set enrichment analysis. (B) The p53 signalling pathway and cell cycle were the critical pathways in response to circSMAD3 silencing. (C) Heatmap showing the apoptosis and cell cycle‐related gene expression in the p53 signalling pathway in RNA sequencing. (D) qPCR showing the detection of the changes in apoptosis and cell cycle‐related genes in the p53 signalling pathway during circSMAD3 silencing. Data are shown as mean ± SD (Student's *t*‐test). (E, F) Western blot to detect proliferative gene (E) and pro‐apoptosis‐related genes (F) expression in circSMAD3 silence. Data are shown as mean ± SD (Student's *t*‐test). (G) Flow cytometry assay to detect the apoptosis of HASMCs during circSMAD3 silencing. Data are shown as mean ± SD (Student's *t*‐test). (H) Chromatin immunoprecipitation‐qPCR used to detect the binding intensity of p53 on its target gene promoters when circSMAD3 is silenced. Data are shown as mean ± SD (Student's *t*‐test). (I) RNA level of p53 isoform in response to circSMAD3 silencing. Data are shown as mean ± SD (Student's *t*‐test). (J) Western blot showing the detection of changes in the protein level of p53 isoform and modification level when circSMAD3 is silenced. Data are shown as mean ± SD (Student's *t*‐test). ns, no significance, ***p<0.001, **p<0.01, *p<0.05.

To elucidate the mechanism through which circSMAD3 inhibited VSMC phenotype switching and proliferation in vitro, thereby attenuating post‐injury neointima formation in vivo, we performed transcriptional sequencing to comprehensively describe transcriptomic changes in circSMAD3 silencing HASMCs. Gene set enrichment analysis showed that the genes modulated by circSMAD3 were mainly enriched in the DNA replication pathway, p53 signalling pathway, and cell cycle (Figure 5A–C). The p53 signalling pathway is critical for cell cycle progression and apoptosis; therefore, we hypothesized that circSMAD3 suppresses proliferation and maintains the contractile state of VSMC by activating the p53 signalling pathway. Thus, we examined the expression of downstream genes modulated by p53 in response to changes in circSMAD3 expression. Consistent with the transcriptional sequencing results, circSMAD3 silence promoted the expression of cell cycle‐related genes (CDK1, CDK2, CCND1, and CCNE2) (Figure 5D,E). However, the upregulation of cell cycle‐related genes induced by PDGF‐BB was significantly reduced by circSMAD3 overexpression (Figure S3A,B). Furthermore, when circSMAD3 was knocked down, apoptosis genes, such as PUMA, p21 and NOX, were downregulated (Figure 5F), whereas circSMAD3 overexpression was inhibited (Figure S3C). Consistent with the changes in pro‐apoptotic genes, the pro‐apoptosis effect of circSMAD3 on HASMCs was also verified using flow cytometry assay during circSMAD3 silencing or overexpression (Figure 5G and Figure S3D).

However, when investigating the role of circSMAD3 in p53, we found that p53 expression was not associated with circSMAD3 at the RNA or protein level, including phosphorylated p53 (Figure 5I,J and Figure S3F,G). Notably, circSMAD3 knockdown obviously declined the transcriptional function of p53 on its target genes, such as p21, PUMA, and NOXA, whereas its overexpression enhanced this function (Figure 5H, Figure S3E). Subsequently, we investigated whether circSMAD3 affected p53 variants. Our results showed that p53γ messenger RNA (mRNA) and protein were downregulated upon circSMAD3 silencing (Figure 5I,J). However, circSMAD3 overexpression promoted the protein level expression of p53γ (Figure S3F,G). Consequently, other variants, such as p53β, Δ40p53, and Δ133p53, had no alterations in response to circSMAD3 changes.

We also designed three small interfering RNA (siRNA) targeted p53γ to investigate whether p53γ mediated the effect of circSMAD3 on VSMC biology. We confirmed that the third siRNA could successfully silence p53γ (Figure S4A,B). Therefore, we selected the third siRNA for further study. p53γ silencing obviously reversed the inhibitory effect on proliferation and pro‐apoptosis effect of circSMAD3 in HASMCs, indicated using the CCK8, EdU, and wound healing assays (Figure S4C–G). Therefore, we conclusively established that circSMAD3 repressed the p53γ signalling pathway, thereby maintaining the differentiated phenotype of VSMCs.

### 2.5 CircSMAD3 interacts with hnRNPA1 to promote its degradation through the ubiquitination‐protease pathway

**FIGURE 6:**
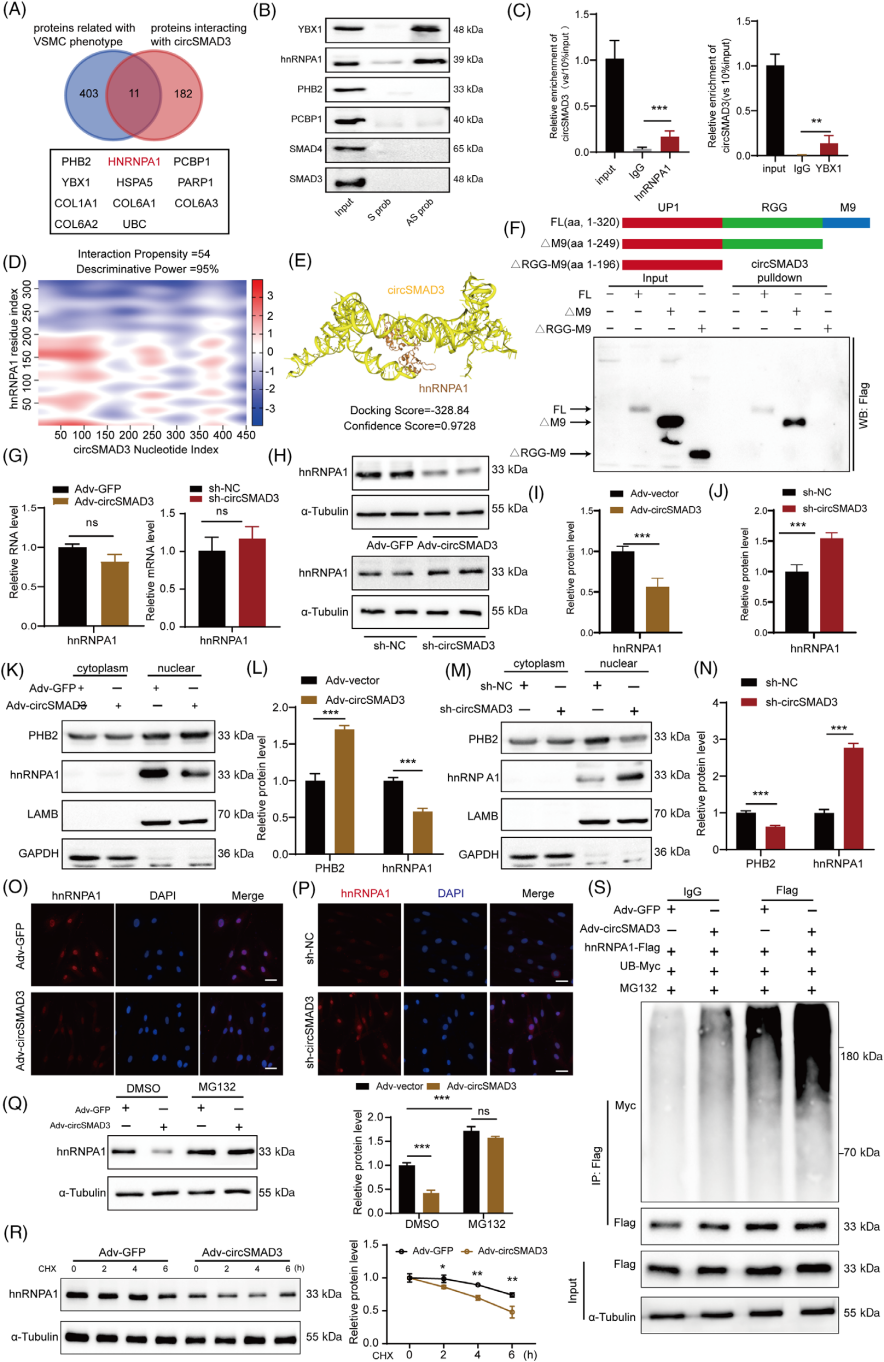
circSMAD3 interacts with hnRNPA1 and promotes its degradation through the protease‐ubiquitination pathway. (A) The 11 proteins associated with VSMC phenotype and interacting with circSMAD3. (B) RNA pull‐down to detect circSMAD3 interacted with hnRNPA1 and YBX1. (C) RIP assay showing the enrichment of circSMAD3 by hnRNPA1 and YBX1. Data are shown as mean ± SD (one‐way ANOVA). (D) Protein‐RNA program‐catRapid predicted the N‐terminal of hnRNPA1 interacting with circSMAD3. (E) HDOCK program for predicting the binding of hnRNPA1 to circSMAD3. (F) Truncated and RNA pull‐down assays demonstrating circSMAD3 interaction with the RGG‐M9 region. (G–J) The changes in hnRNPA1 in RNA (G) and protein levels (H–J) when circSMAD3 is overexpressed or silenced. Data are shown as mean ± SD (Student's *t*‐test). (K–N) Cell fraction assay showing the detection of the changes in hnRNPA1 in response to circSMAD3 overexpression (K, L) and silencing (M, N) using Western blot. Data are shown as mean ± SD (Student's *t*‐test). (O, P) Immunofluorescence assay showing the detection of the changes in hnRNPA1 when circSMAD3 is overexpressed (O) or silenced (P). Scale bar = 50 μm. (Q, R) Western blot showing the changes in hnRNPA1 protein in response to circSMAD3 overexpression and MG132 (20 ng/mL) or CHX (20 ng/mL). Data are shown as mean ± SD (two‐way ANOVA) in (Q) and mean ± SD (Student's *t*‐test) in (R). (S) Ubiquitin assay to demonstrate that circSMAD3 promoted the level of ubiquitin on hnRNPA1. ns, no significance, ***p<0.001, **p<0.01, *p<0.05

The interaction of circRNAs with proteins is a classical mechanism through which they function.^34^ We identified a set of proteins among the products of circRNA pull‐down in HASMCs using mass spectrometry. We also comprehensively collected a set of proteins associated with VSMC physiology from the gene ontology database and relevant literature and found 11 proteins present in both datasets (Figure 6A). Only hnRNPA1 and Y‐Box Binding Protein 1 (YBX1) interacted with circSMAD3 through RNA pull‐down and ribonucleoprotein immunoprecipitation (RIP) assays (Figure 6B,C). hnRNPA1, an RNA‐binding protein, enhances arterial injury‐induced neointima formation by modulating pre‐RNA splicing.^35^ Therefore, we considered hnRNPA1. Bioinformatics analysis using catRapid^36^ and HDOCK^37^ demonstrated that the N‐terminal part of hnRNPA1 could bind to circSMAD3 (Figure 6D,E). Therefore, we truncated this protein into three parts with a 3x FLAG tag according to its domains, as shown in Figure 5F. The RNA pull‐down assay result showed that circSMAD3 mainly interacted with the RGG‐M9 domain of hnRNPA1 (Figure 6F).

Furthermore, we explored the molecular consequences of the interaction between circSMAD3 and hnRNPA1. CircSMAD3 overexpression and silencing markedly decreased and increased hnRNPA1 protein levels, respectively (Figure 6H–J). However, we did not observe a reduction in hnRNPA1 transcription (Figure 6G). Reportedly, hnRNPA1 primarily exists in the nucleus to perform its splicing function in pre‐RNA. Therefore, we speculated that the influence of circSMAD3 on hnRNPA1 mainly occurs in the nucleus. Cytoplasmic and nuclear fraction separation and immunofluorescence assays showed that the hnRNPA1 protein levels in the nuclear fraction were significantly downregulated when circSMAD3 was overexpressed, whereas circSMAD3 silencing stabilized hnRNPA1 protein level within the nucleus (Figure 6K–P). Prohibitin 2 (PHB2), a protein that reportedly binds with hnRNPA1 to repress its splicing function, was significantly upregulated in response to circSMAD3 overexpression in the nucleus and downregulated when circSMAD3 was silenced (Figure 6K–N). MG132, an inhibitor of the protease‐ubiquitination pathway, significantly reversed the circSMAD3‐induced reduction in hnRNPA1 protein levels. However, cycloheximide (CHX) as a protein synthesis inhibitor could not achieve this (Figure 6Q,R). Moreover, the immunoprecipitation (IP) assay showed that circSMAD3 overexpression dramatically promoted the ubiquitin level of hnRNPA1 (Figure 6S). These results suggest that circSMAD3 modulated hnRNPA1 expression through the protease‐ubiquitination pathway.

### 2.6 CircSMAD3 enhances the binding of hnRNPA1 with E3 ligase WDR76

**FIGURE 7:**
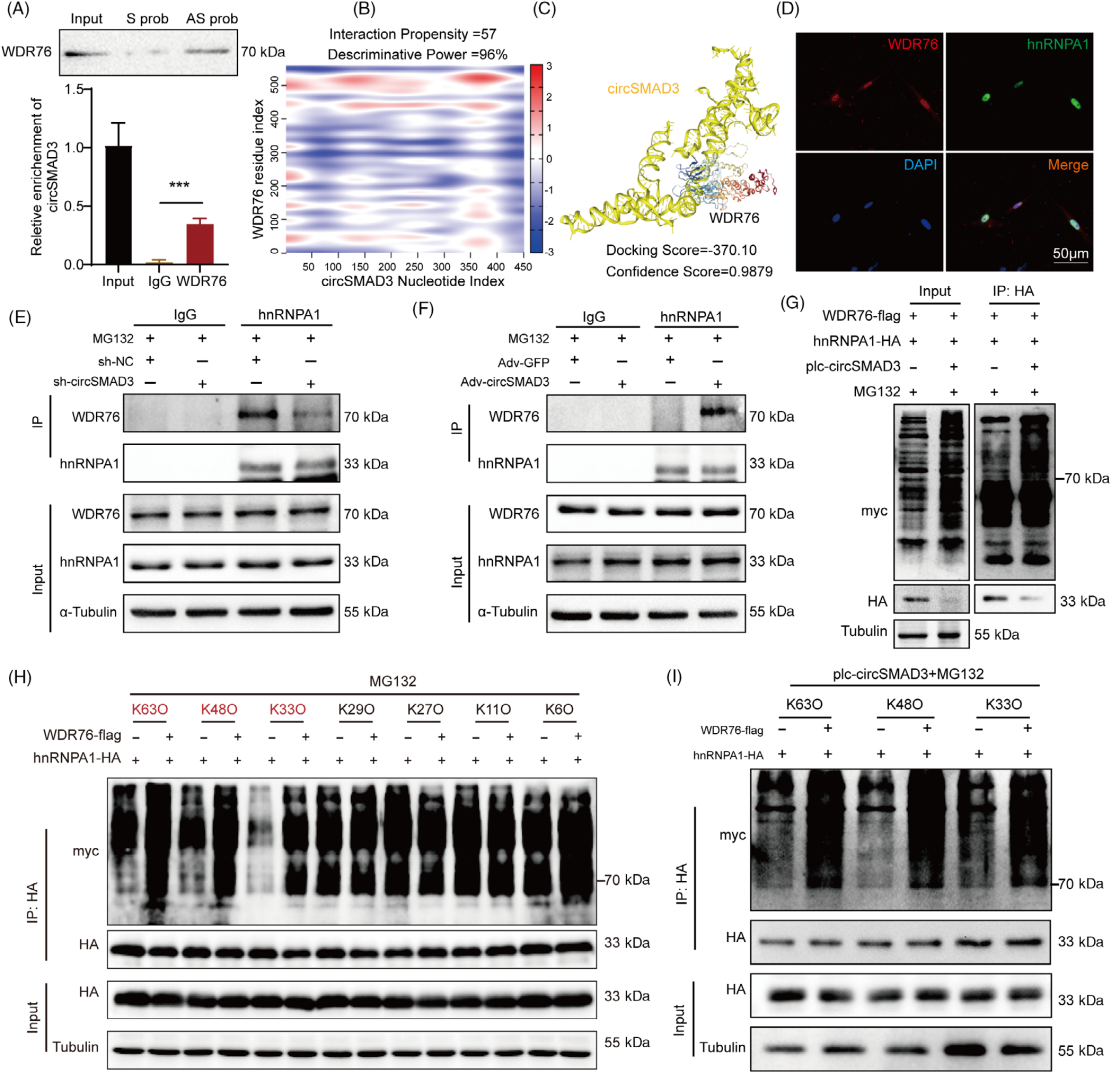
circSMAD3 promoted the ubiquitination degradation of hnRNPA1 by E3 ligase WDR76. (A) RIP (upper) and RNA pull‐down (lower) assays showing the detection of the binding of hnRNPA1 to circSMAD3. Data are shown as mean ± SD (Student's *t*‐test). (B, C) CatRapid and HDOCK predicted that the N‐terminus of hnRNPA1 interacts with circSMAD3. (D) Immunofluorescence assay showing the detection of the co‐localization of hnRNPA1 with WDR76 in HASMCs. Scale bar = 50 μm. (E, F) IP assay to detect the interaction between hnRNPA1 and WDR76 upon circSMAD3 silencing (E) and overexpression (F). (G) IP assay showing that circSMAD3 overexpression promoted the ubiquitination level of hnRNPA1 in HEK293T cells. (H) Western blot showing the ubiquitination levels of hnRNPA1 after Flag‐WDR76 overexpression and in response to MG132 treatment in HEK293T cells co‐transfected with hemaglutinin antigen (HA)‐tagged hnRNPA1 and Myc‐tagged ubiquitin (K63O, K48O, K33O, K29O, K27O, K11O, and K6O) constructs. K63O indicates ubiquitin, in which only lysines‐K63 were obtained. (I) Western blot showing the ubiquitination levels of hnRNPA1 after circSMAD3 overexpression and in response to MG132 treatment in HEK293T cells co‐transfected with HA‐tagged hnRNPA1, Flag‐WDR76, and Myc‐tagged ubiquitin (K63O, K48O, and K33O) constructs. ***p<0.001.

Given the degradation function of circSMAD3 in hnRNPA1 through the protease‐ubiquitination pathway, it is unclear whether ubiquitin‐related proteins are involved. Based on the mass spectrometry results, there are three ubiquitin‐related molecules, including WDR76, UBA52, and FAU. UBA52 and FAU are members of the ubiquitin family, which often participate in the progress of protein degradation by binding to protein directly. Only WDR76 belongs to E3 ubiquitin ligase. Therefore, we selected WDR76 for further study. Using RNA pull‐down and RIP assays, we found that WDR76 could interact with circSMAD3 (Figure 7A). We then hypothesized that circSMAD3 functions as a scaffold, enhancing hnRNPA1's binding to the E3 ligase WDR76, thereby mediating the degradation of hnRNPA1. Consequently, bioinformatic methods predicted a strong interaction between circSMAD3 and WDR76 (Figure 7B,C), and the co‐localization analysis revealed that hnRNPA1 and WDR76 existed in the nucleus (Figure 7D). Furthermore, we confirmed the binding of circSMAD3 to WDR76. Notably, circSMAD3 overexpression enhanced the interaction between hnRNPA1 and WDR76, whereas circSMAD3 silencing abrogated this interaction (Figure 7E,F). Reciprocally, circSMAD3 overexpression facilitated the WDR76‐induced ubiquitin level of hnRNPA1 (Figure 7G). Further IP assays showed that WDR76 robustly promoted K63‐, K48‐, and K33‐linked ubiquitination of hnRNPA1 (Figure 7H), which was significantly enhanced by circSMAD3 overexpression (Figure 7I). Therefore, these data suggest that circSMAD3 functions as a scaffold that facilitates the degradation of hnRNPA1 depending on ubiquitination by improving the binding of hnRNPA1 to the E3 ubiquitin ligase WDR76.

### 2.7 CircSMAD3 modulated the alternative splicing of p53 pre‐RNA by hnRNPA1

**FIGURE 8:**
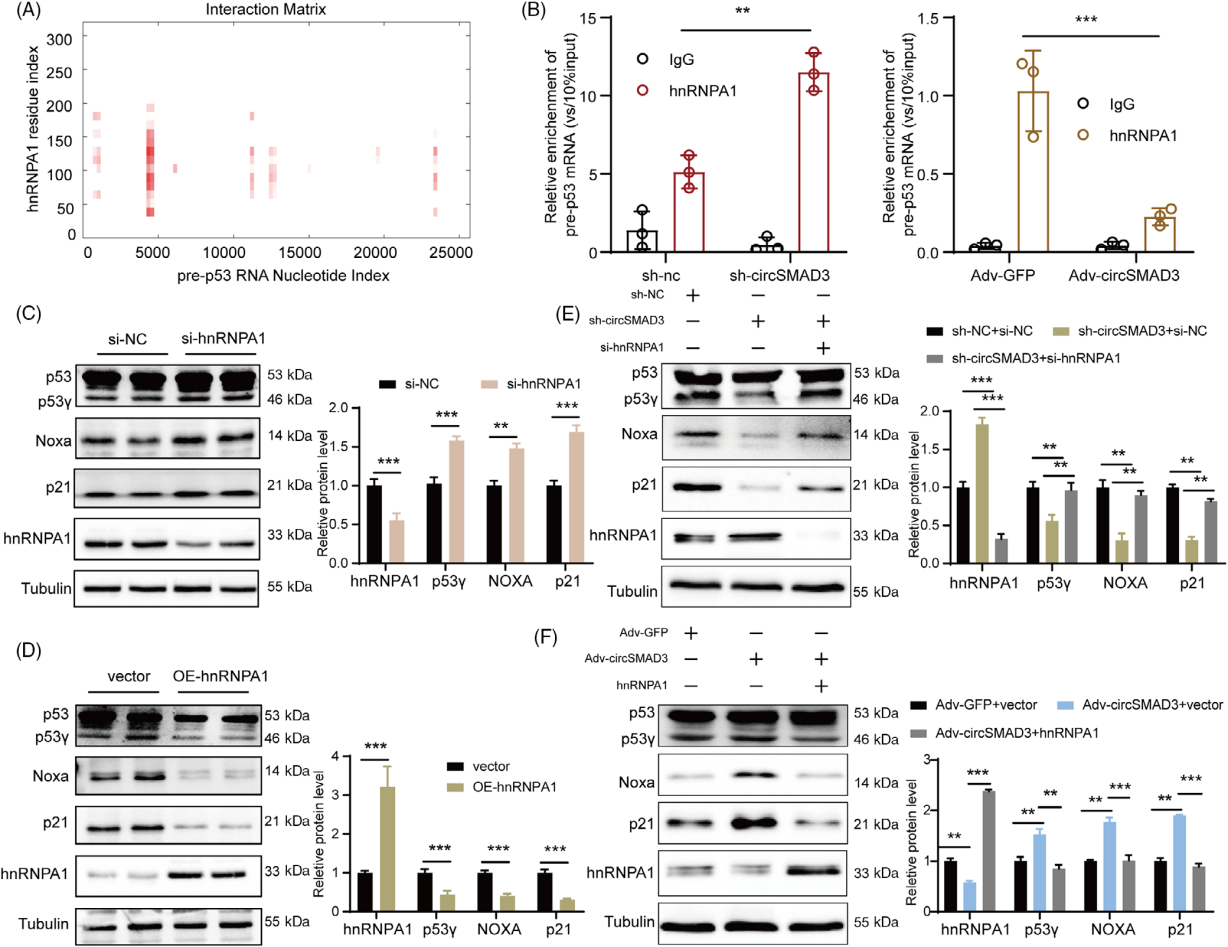
circSMAD3 modulated p53 precursor RNA splicing by hnRNPA1. (A) Protein‐RNA program‐catRapid predicted the binding region of pre‐p53 with hnRNPA1. (B) RIP assay showing the enrichment of pre‐p53 by hnRNPA1 and the enhancement of this interaction by circSMAD3 silencing and overexpression. Data are shown as mean ± SD (two‐way ANOVA). (C) hnRNPA1 silencing significantly promoted the protein level expression of p53γ and its target genes. Data are shown as mean ± SD (Student's *t*‐test). (D) hnRNPA1 overexpression significantly declined the protein level expression of p53γ and its target genes. Data are shown as mean ± SD (Student's *t*‐test). (E) The silencing of hnRNPA1 reversed the downregulation of p53γ and its target genes induced by circSMAD3 silencing. Data are shown as mean ± SD (one‐way ANOVA). (F) The overexpression of hnRNPA1 reversed the upregulation of p53γ and its target genes induced by circSMAD3 overexpression. Data are shown as mean ± SD (one‐way ANOVA). ***p<0.001, **p<0.01.

hnRNPA1 is an RNA‐binding protein that is critical in pre‐RNA processing and splicing in the nucleus as a core member of the hnRNP complex.^38^, ^39^ Therefore, we hypothesized that circSMAD3 modulates p53 pre‐RNA splicing through the splicing factor hnRNPA1. We found many sites in p53 pre‐RNA that interacted with hnRNPA1 using the bioinformatics method catRapid (Figure 8A). Additional evidence revealed that p53 pre‐RNA was significantly enriched by hnRNPA1, as detected using the RIP assay, which was significantly promoted by circSMAD3 silencing and decreased by circSMAD3 overexpression (Figure 8B).

Furthermore, we changed hnRNPA1 expression in HASMCs to investigate the effect of hnRNPA1 splicing on p53 pre‐RNA. hnRNPA1 knockdown promoted the expression of p53γ and apoptosis‐related proteins, whereas overexpression of hnRNPA1 decreased (Figure 8C,D). Additionally, hnRNPA1 silencing abolished the decrease in the protein level of p53γ and its target genes induced by circSMAD3 knockdown (Figure 8E). However, hnRNPA1 overexpression rescued the protein level increase of p53γ and apoptosis‐related genes caused by circSMAD3 upregulation (Figure 8F). Therefore, these results demonstrate that circSMAD3 modulates the splicing process of p53 pre‐RNA mediated by hnRNPA1.

## 3 DISCUSSION

circRNAs have attracted significant attention for developing novel drug therapies because of their safety profile and low immunogenicity. Efficient delivery systems have also accelerated this process significantly. Therefore, elucidating the underlying mechanisms of circRNAs in pathophysiology is essential for developing novel therapeutic targets. Here, we identified a novel circRNA, circSMAD3, which significantly affects VSMC proliferation and phenotype switching in vitro and in vivo. Mechanistically, circSMAD3 interacts with hnRNPA1 to enhance the binding of hnRNPA1 to E3 ligase WDR76, subsequently promoting the ubiquitination degradation of hnRNPA1 to suppress its splicing function on p53 pre‐RNA and eventually activating the p53γ signalling pathway.

A notable finding is the modulation of circSMAD3 to splice p53 pre‐RNA, promote p53γ expression, and enhance the signal transduction of the p53 signalling pathway. In total, 14 p53 isoforms have been identified because of the alternative splicing of p53 pre‐RNA, among which p53β, p53γ, Δ40p53, and Δ133p53 have been extensively explored to play their functions through the p53 signalling pathway.^40^ p53β and p53γ enhance the transcriptional modulation of p53 to its target genes by facilitating the binding of p53 to the promotor region; however, this effect is not observed across all p53‐inducible promotors.^41^ Additionally, Δ40p53 impaired the growth suppression mediated by p53,^42^, ^43^ whereas Δ133p53 inhibited the apoptosis induced by p53.^44^, ^45^ However, the alternative splicing of p53 has rarely been studied in patients with vascular diseases so far, except for circEsyt2 recruiting polyC‐binding protein 1 which was used to mediate p53 splicing and facilitate p53β expression.^18^ This study found that circSMAD3 participates in the p53 signalling pathway through the transcriptional analysis of HASMCs. Therefore, we focused on p53, which is a core factor in this pathway. Further investigation confirmed that, even after phosphorylation, circSMAD3 did not alter the transcriptional or post‐transcriptional level of p53 expression. We investigated whether the alternative isoforms of p53 participate in the circSMAD3‐mediated process. Fortunately, p53γ, rather than other isoforms, had a strong relationship with circSMAD3. Therefore, we speculated that circSMAD3 maintained VSMC homeostasis by activating the p53γ signalling pathway.

Another critical finding in this study was the interaction of circSMAD3 with hnRNPA1 to promote its degradation by ubiquitin, which is a splicing factor associated with pre‐mRNAs in the nucleus and influences pre‐RNA processing. In vascular diseases, hnRNPA1 significantly affects pyruvate kinase M (PKM) 1/2 RNA splicing and reversion from PKM1 to PKM2, ultimately enhancing glycolysis and VSMC phenotype switching. Additionally, we found that hnRNPA1 binds to circSMAD3 by combining the RNA pull‐down products in HASMCs with proteins associated with VSMC physiology. Further truncation experiments confirmed that the RGG‐M9 domain in hnRNPA1 was mainly bound to circSMAD3, consistent with PHB2, a direct hnRNPA1 repressor. Additionally, circSMAD3 expression changes synchronously with PHB2 expression in the nucleus, suggesting a synergistic effect between circSMAD3 and PHB2 in modulating VSMC physiology. We also explored the relationship between circSMAD3 and hnRNPA1 and found that circSMAD3 promoted hnRNPA1 degradation through the ubiquitination‐protease pathway using the E3 ligase WDR76. Ubiquitination assays also showed that circSMAD3 promoted the K63‐, K48‐, and K33‐linked ubiquitination degradation of hnRNPA1 by enhancing the binding of hnRNPA1 to the E3 ligase WDR76 in HASMCs.

We speculated whether hnRNPA1 participates in p53 pre‐RNA splicing, considering its role in the alternative splicing of pre‐RNA. A RIP assay confirmed that the effect of circSMAD3 on p53 pre‐RNA splicing was modulated by hnRNPA1. hnRNPA1 silencing in HASMCs significantly promoted p53γ expression in the RNA and protein levels, whereas other isoforms had no changes. In contrast, hnRNPA1 overexpression was markedly suppressed. p53γ upregulation induced by circSMAD3 overexpression was immensely abolished by hnRNPA1 overexpression, indicating that hnRNPA1 mediated the process of circSMAD3 modulating p53 pre‐RNA splicing.

In the pathological process of vascular diseases, phenotype switching and apoptosis of VSMC co‐occur. In vascular injury, glucose‐6‐phosphate dehydrogenase (G6PD) inhibited VSMC apoptosis, promoted phenotype switching, and accelerated vascular neointima hyperplasia by reducing voltage‐dependent anion‐selective channel protein 1 (VDAC1) oligomerization.^46^ CircRNA circEsyt2 also enhanced VSMC proliferation and migration and inhibited apoptosis and differentiation.^18^ However, KDM1A silencing reversed the inhibitory effect of VSMC apoptosis induced by angiotensin II.^47^ The long non‐coding RNA H19 silencing significantly suppressed HASMC apoptosis and phenotype switching in patients with abdominal aortic aneurysms.^48^ However, BAF60c knockdown in VSMCs resulted in loss of contractile phenotype, increased VSMC inflammation, and apoptosis.^49^ In this study, we also illustrated the changes of phenotype switching and apoptosis of VSMC in response to circSMAD3 by p53 pre‐RNA splicing, which has not been reported previously.

This study has some limitations. First, we did not confirm the function of circSMAD3 using circSMAD3 transgenic or gene knockout mice, which could better verify the function of circSMAD3 in vascular biology. Second, we tried to establish the relationship between circSMAD3 and its parent gene SMAD3. Unfortunately, circSMAD3 had little effect on SMAD3 expression in the transcriptional and protein levels, even its phosphorylated modification. Furthermore, RNA pull‐down and RIP assays verified that circSMAD3 was barely bound with SMAD3. However, circSMAD3 has little effect on the distribution of phosphorylated SMAD3 in the cytoplasm and nucleus. Consequently, the effect was not enough to attract our attention to further investigate the underlying mechanism. Third, hnRNPA1 greatly affected PKM pre‐RNA splicing and then mediated glucose metabolic reprogramming. We also found some effects of circSMAD3 in glucose metabolism through RNA sequencing. However, this is not the focus of our present work; therefore, we did not explore it in depth.

In conclusion, circSMAD3 suppressed VSMC proliferation and phenotype switching. It prevented neointima formation from promoting the interaction of hnRNPA1 with WDR76, which facilitates hnRNPA1 degradation depending on ubiquitination and subsequently activates the p53γ signalling pathway. This may be a novel therapeutic strategy for vascular proliferative diseases.

## 4 MATERIALS AND METHODS

### 4.1 Cell culture and treatment

A HASMC was purchased from Anwei Biotechnology Co., Ltd (Shanghai, China) and cultured in ICell Primary Smooth Muscle Cell Low Serum Culture System (Anwei Biotechnology Co, Shanghai, China) in a humidified atmosphere at 37°C with 5% carbon dioxide (CO_2_). mASMCs were isolated from adult mice aged 8 weeks and cultured in Dulbecco's Modified Eagle's Medium (DMEM) containing 10% foetal bovine serum (FBS, GIBCO, Brazil) in a humidified atmosphere at 37°C with 5% CO_2_. We applied passages 4–15 of HASMCs and mASMCs in this study. The siRNAs targeting circSMAD3, hnRNPA1 and p53γ were dissolved in enzyme‐free water at 50uM. The sequence of siRNAs is listed in Table S3.

### 4.2 RNA isolation, quantitative reverse transcriptase‐PCR, and PCR

Total RNA was extracted using TRIzol reagent (Vazyme, Nanjing, China), as previously described. The purity and concentration of the total RNA were determined using a NanoDrop ND‐2000 (NanoDrop Thermo, Wilmington, DE, USA). Furthermore, the reverse transcription of circRNAs was performed using the Takara System (Dalian, China). Real‐time polymerase chain reaction (PCR) was performed using the Vazyme System (Vazyme, Nanjing, China) on a 7900HT FAST qPCR System (Life Technologies, Carlsbad, CA, USA). The level of genes was calculated based on the cycle threshold (Ct) values compared with a reference gene using the formula 2^−ΔΔCt^. Glyceraldehyde‐3‐phosphate dehydrogenase (GAPDH) mRNA was used as a reference for mRNA and circRNA expressions. PCR was performed following the manufacturer's instructions (NEO, Japan). The details of the primers used are listed in Tables S1 and S2.

### 4.3 Protein extraction and Western blotting

Briefly, the cells were lysed in ice‐cold IP lysis buffer (Beyotime, Shanghai, China) and centrifuged at 12,000 × *g* for 15 min at 4°C. The supernatant was collected, and the protein concentration was analysed using a NanoDrop ND‐2000 (Thermo, Wilmington, DE, USA). Furthermore, the protein levels were normalized by probing the same blots with antibodies against alpha‐tubulin (Abclonal, Wuhan, China) or GAPDH (Abclonal, Wuhan, China). The primary antibody for western blot in this study included α‐SMA (A17910, Ablonal, China), TAGLN (ab10135, Abcam, USA), CNN1 (A3734, Abclonal, China), CDK1 (19532‐1‐AP, Proteintech, China), CDK2 (10122‐1‐AP, Proteintech, China), CCNE2 (11935‐1‐AP, Proteintech, China), CCND1 (60186‐1‐Ig, Abclonal, China), p21 (10355‐1‐AP, Proteintech, China), Puma (55120‐1‐AP, Proteintech, China), NOXA (A9801, Abclonal, China), hnRNPA1 (11176‐1‐AP, Proteintech, China), WDR76 (25528‐1‐AP, Proteintech, China), YBX1 (A3534, Abclonal, China), PHB2 (66424‐1‐Ig, Proteintech, China), PCBP1 (14523‐1‐AP, Proteintech, China), SMAD3 (ab40854, Abcam, USA), SMAD4 (ab40759, Abcam, USA), p53 ((DO‐1): sc‐126, Santa, USA), p53 (A10610, Abclonal, China), and p‐p53 (28961‐1‐AP, Abclonal, China).

### 4.4 Fluorescence in situ hybridization

A Digoxigenin‐labelled probe specific for the junction site of circSMAD3 was designed and synthesized by Sangon bio (Shanghai, China). Antibody against Digoxigenin was purchased from Abcam (ab51949, MA, USA). Tyramide signal amplification system was purchased from Servicebio (Wuhan, China). The protocol was performed following the manufacturer's instructions. The signals of the probe were detected, and the images were acquired on a Lei TCS SP8 laser scanning confocal microscope (Leica Microsystems, Mannheim, Germany).

### 4.5 Immunofluorescence analysis

HASMCs and mASMCs were washed with phosphate‐buffered saline (PBS), fixed in 4% paraformaldehyde, permeabilized with 0.5% Triton X‐100 (Beyotime, Shanghai, China) in PBS for 15 min, and incubated with 5% bovine serum albumin for 1 h at room temperature. Subsequently, the cells were incubated with primary antibodies (1:100) at 4°C overnight. The primary antibodies used included anti‐α‐SMA (1:200, Abclonal, China), anti‐hnRNPA1 (1:200, Proteintech, China), and anti‐WDR76 (1:200, Proteintech, China) antibodies. After the overnight incubation, the samples were incubated with secondary antibodies (FITC mouse, CY3 rabbit; Servicebio, Wuhan, China) at 1:500 for 1 h, washed thrice with PBS, and then incubated with 4′,6‐Diamidino‐2‐phenylindole (DAPI) for 15 min. The images were captured using a fluorescence microscope (Axio Imager 2, Zeiss, Germany).

### 4.6 Cell area measure

First, HASMC and mASMC were seeded onto glass coverslips in 24‐well plates. The cells were washed twice with PBS, fixed in 4% paraformaldehyde, and permeabilized with 0.5% Triton X‐100 for 15 min. Subsequently, the samples were incubated with 0.1% phalloidin (Abclonal, Wuhan, China) for 30 min at 37°C and incubated with DAPI at 1:100 dilution for 15 min. Coverslips were placed onto glass slides using a mounting medium, and the images were captured using a fluorescence microscope (Axio Imager 2, Zeiss, Germany). This process was repeated thrice.

### 4.7 Chromatin immunoprecipitation assay (ChIP)

Chromatin immunoprecipitation assay was conducted to verified the binding intensity of p53 on its target genes (P21, PUMA and NOXA) promotor region with circSMAD3 overexpression or silence according to the ChIP Assay Kit (Biyotime, Wuhan, China). In brief, the cells were crosslinked by 1% Formaldehyde, neutralized by 2 mg/mL glycine, then washed by PBS and then lysed by lysis buffer. The cell lysis was sonicated to break DNA into pieces and then centrifuged. The supernatants were incubated with antibody against p53 overnight with shaking. The complex was washed according to the protocols and purified by DNA purification kit (Biyotime, Wuhan, China). Binding intensity of p53 on its target gene promotors was identified by qPCR. The primers of target gene promotor region are listed in Table S4.

### 4.8 Pull‐down assay with biotinylated circSMAD3 probe

The biotinylated circSMAD3 probe was synthesized using AokeBoTai (Wuhan, China), and the oligo‐probe was used as a control. This assay was performed as previously described. The circSMAD3 probe was incubated with streptavidin magnetic beads (Life Technologies, CA, USA) at room temperature for 1 h to generate probe‐coated beads. The cell lysates were incubated with the probe‐coated beads at room temperature for 2 h. Furthermore, the complexes were washed twice and divided into two parts for RNA and protein extraction. The bound RNA in the pull‐down materials was extracted using TRIzol reagent and analysed using quantitative reverse transcription‐PCR (qRT‐PCR). Finally, the proteins in the complexes were detected using Western blotting. The probe sequence used in the study is shown in Table S5. The proteins identified in HASMC by Mass spectrum are shown in Table S6. The proteins related to VSMC phenotype in GO database and literatures are shown in Table S7.

### 4.9 RNA immunoprecipitation

RIP was conducted using the Magna RIP Kit (Millipore, Billerica, MA, USA) following the manufacturer's instructions. Antibodies against hnRNPA1, WDR76, YBX1, and immunoglobulin G used for RIP were purchased from Proteintech Bio. The cells were washed twice with PBS and lysed using IP lysis buffer containing RNase and protease inhibitors. The supernatants were collected after centrifugation for further analysis. Furthermore, the antibodies were incubated with protein A/G beads for 1 h at room temperature, and the complexes of antibody and beads were subsequently incubated with cell supernatant overnight at 4°C. RNA in the complex was extracted using TRIzol reagent. Finally, qRT‐PCR was used to evaluate the enrichment of circSMAD3 and pre‐p53.

### 4.10 Construction of carotid arterial injury model

C57B/J mice were purchased from Gempharmatech Co., Ltd (Jiangsu, China) aged 7 weeks. Eight‐week‐old C57BL/6J mice was injected with circSMAD3 overexpression or sh‐RNA adeno‐associated virus at a titer of 5 × 1011 v.g./mL (100μL per mouse) by tail vein. After 3 weeks, the mice were induced to arterial injury model on the right common carotid arterial by wire. Briefly, the mice were anesthetized with 1% pentobarbital (Sigma, USA), and fixed on the plate. The carotid skin and connective tissue were cut off and separated to clearly expose the common, internal and external carotid arterial under a dissecting microscope. Then the proximal common carotid arterial and internal carotid arterial were clipped for the temporary blockage of blood flow and the distal external carotid arterial was ligated with 7‐0 silk sutures. Then, a wire (0.38 mm) was introduced from external carotid arterial into the common arterial lumen and withdrawn 5 times. After that, the proximal distal carotid arterial was ligated to seal the breach. The proximal common carotid arterial and internal carotid arterial were subsequently released to restore blood flow. The operated mice were kept warm and revived on an electric blanket. After four weeks, the carotid arteries were collected. All mice were maintained and studied using protocols following the National Institutes of Health Guide for the Care and Use of Laboratory Animals and approved by the Committee on the Ethics of Animal Experiments of the Animal Research Committee of Tongji Medical College, Huazhong University of Science and Technology ([2022] IACUC Number3836).

### 4.11 Statistical analysis

All statistical analyses were performed using IBM SPSS Statistics for Windows, version 22 (IBM Corp., Armonk, NY, USA) and GraphPad Prism 8 (GraphPad Software Inc., MA, USA). Student's *t*‐test and one‐ or two‐way analysis of variance were used for multiple groups experiment statistical analysis. All data from three independent experiments are presented as the mean ± standard deviation or mean ± standard error of the mean. Statistical significance was set at *p* < 0.05.

## AUTHOR CONTRIBUTIONS

Hu Ding and Jiangtao Yan designed the study and analysed the data. Shuai Mei, Xiaozhu Ma and Li Zhou conducted the experiments. Shuai Mei, Xiaozhu Ma, Qidamgai Wuyun, and Ziyang Cai analysed the data and provided experimental materials. Shuai Mei and Hu Ding drafted the manuscript. All the authors have read and approved this manuscript.

## FUNDING INFORMATION

This study was supported by grants from the National Natural Science Foundation of China (grant numbers: 82170392, 82170348, and 81974047), the National Key Research and Development Program of China (grant numbers: 2021YFC2500600 and 2021YFC2500604), and the Hubei Key Research and Development Program (grant number: 2021BCA121).

## CONFLICT OF INTEREST STATEMENT

The authors declare that they have no competing interests.

## Supporting information


Data S1.

## Data Availability

The RNA‐sequencing data have been uploaded into the GEO database and released in collaboration with the NCBI Bioproject (accession number: PRJNA1016164). The data that support the findings of this study are available from the corresponding author upon reasonable request.
