# Supplementary material for: CircSMAD3 represses VSMC phenotype switching and neointima formation via promoting hnRNPA1 ubiquitination degradation
Source: Cell Prolif. 2024 Sep 1;58(1):e13742. doi: 10.1111/cpr.13742 (PMC11693546; doi:10.1111/cpr.13742)
Supplement: Supplementary file 1 — Data S1. [file CPR-58-e13742-s001.docx]

# circSMAD3 regulates VSMC phenotype switching and represses neointima formation through promoting hnRNPA1 ubiquitination degradation

Shuai Mei^1,2^, Xiaozhu Ma^1,2^, Li Zhou^1,2^, Qidamugai Wuyun^1,2^, Ziyang Cai^1,2^, Jiangtao Yan^1^, Hu Ding^1,2^

^1^Division of Cardiology, Departments of Internal Medicine, Tongji Hospital, Tongji Medical College, Huazhong University of Science and Technology, Wuhan 430030, People’s Republic of China

^2^Hubei Key Laboratory of Genetics and Molecular Mechanisms of Cardiological Disorders, Wuhan 430030, China

Corresponding Authors:

Hu Ding, MD, PhD or Jiangtao Yan, MD, PhD

Division of Cardiology, Departments of Internal Medicine, Tongji Hospital, Tongji Medical College, Huazhong University of Science and Technology,

1095# Jiefang Ave, Wuhan 430030, People’s Rep. of China

Phone & Fax: 86-27-8366-3280

Email: huding@tjh.tjmu.edu.cn or jtyan@tjh.tjmu.edu.cn

**Supplementary materials include:**

Supplementary Materials and Methods

Supplementary Table 1-7

Supplementary Figure 1-4

## Supplementary Materials and Methods

**Cell culture and treatment**

Human primary aortic smooth muscle cell (HASMC) was purchased from Anwei Biotechnology Co., Ltd (Shanghai, China) and cultured in ICell Primary Smooth Muscle Cell Low Serum Culture System (Anwei Biotechnology Co, Shanghai, China) in a humidified atmosphere at 37°C with 5% CO2. Mouse primary aortic smooth muscle cells (mASMC) were isolated from adult mice aged 8 weeks and was cultured in Dulbecco’s Modified Eagle’s Medium (DMEM) containing 10% fetal bovine serum (FBS, GIBCO, Brazil) in a humidified atmosphere at 37°C with 5% CO2. Passages 4-15 of HASMC and mASMC were applied to this study.

For the treatment by cytokines to VSMC, TGFβ1 and PDGF-BB (MCE, USA) was dissolved in ultrapure water as the final concentration of 10mg/L and 20mg/L, respectively. When stimulated the starved cells for another 24 hours, those cytokines concentration was 10ug/L or 20ug/L, respectively.

**RNA isolation, quantitative RT-PCR and PCR**

Total RNA was extracted using RNA iso reagent (Takara, DaLian, China) as previous mentioned. Purity and quantity of total RNA was detected by NanoDrop ND-2000 (NanoDrop Thermo, Wilmington, DE, USA). Reverse transcription of RNA performed according to PrimeScript™ RT reagent Kit (RR037A, Takara). The quantification of RNA relied on Real time PCR using Vazyme system (Vazyme, Nanjing, China) on a 7900HT FAST Real-time PCR System (Life Technologies, Carlsbad, CA). The expression of genes was calculated based on the cycle threshold (Ct) values compared to a reference gene using the formula 2^-ΔΔCt^. GAPDH mRNA and U6 snRNA were used as references for mRNA and circRNA. Polymerase chain reaction (PCR) was performed according to PrimeSTAR^®^ GXL DNA Polymerase kit (R050A, Takara). The details of primers were listed in **Supplementary Table 1** and **Supplementary Table 2**.

**Western blot**

The cells were lysed in ice-cold IP lysis buffer (Beyotime, Shanghai, China), incubated with shaking in 4 °C for 20min, and then centrifuged for 15min (12,000×g, 4 °C). The supernatant was collected, and the concentration of protein was detected by NanoDrop ND-2000 (Thermo, Wilmington, DE, USA). The protein levels were normalized by probing the same blots with antibody against α-Tubulin (Abclonal, Wuhan, China) or GAPDH (Abclonal, Wuhan, China). The primary antibody for western blot in this study included α-SMA (A17910, Ablonal, China), TAGLN (ab10135, Abcam, USA), CNN1 (A3734, Abclonal, China), CDK1 (19532-1-AP, Proteintech, China), CDK2 (10122-1-AP, Proteintech, China), CCNE2 (11935-1-AP, Proteintech, China), CCND1 (60186-1-Ig, Abclonal, China), p21 (10355-1-AP, Proteintech, China), Puma (55120-1-AP, Proteintech, China), NOXA (A9801, Abclonal, China), hnRNPA1 (11176-1-AP, Proteintech, China), WDR76 (25528-1-AP, Proteintech, China), YBX1 (A3534, Abclonal, China), PHB2 (66424-1-Ig, Proteintech, China), PCBP1 (14523-1-AP, Proteintech, China), SMAD3 (ab40854, Abcam, USA), SMAD4 (ab40759, Abcam, USA), p53 ((DO-1): sc-126, Santa, USA), p53 (A10610, Abclonal, China), p-p53 (28961-1-AP, Abclonal, China).

**Adeno-associated virus type 9, adenovirus package and infection**

For in vitro experiment, circSMAD3 overexpression adenovirus and sh-circSMAD3 adenovirus were packaged by HanBio (Shanghai, China) and WZBio (Shandong, China). When being infected, the HASMC and mASMC was infected by circSMAD3 overexpression adenovirus, sh-circSMAD3 adenovirus and their control adenovirus with 100 MOI. For in vivo experiment, circSMAD3 overexpression and sh-circSMAD3 adeno-associated virus type 9 with Tagln promotor were packaged by WZBio (Shandong, China), and infected the C57BL/6J mice at a titer of 5 × 10^11^ v.g./mL (100μL per mouse) by tail vein.

**Plasmid construction, and siRNA transfection**

The siRNAs targeting circSMAD3, hnRNPA1 and p53γ were synthesized by Aokebotai Bio (Wuhan, China) and transfected at a final concentration of 100nM into cells diluted in Opti-MEM (Gibco, Thermo Fisher Scientific). The sequence of siRNAs was listed in **Supplemental Table 3**. hnRNPA1-3xFlag, hnRNPA1-HA, WDR76-3xFlag overexpression plasmid was constructed by Aokebotai Bio (Wuhan, China). For the performance of hnRNPA1 overexpression in HASMC, the plasmid of 3x flag-hnRNPA1 was transfected by Lipofectamine3000 reagent (Gibco, Thermo Fisher Scientific) following the manufacturer’s instructions.

In ubiquitin experiments, circSMAD3 overexpression plasmid was constructed by Geneseed bio (Guangzhou, China). The cDNA sequence of circSMAD3 was synthesized and inserted into pLC5 vector plasmid. Various of pCMV-Myc-ubiquitin (WT, K63O, K48O, K33O, K29O, K27O, K11O, K6O) were purchased from Aokebotai Bio (Wuhan, China). Those plasmids were transfected into 293HEK cells for 48 hours following MG132 (MCE, China) stimulation at 20μg/l.

**Fluorescence in situ hybridization (FISH)**

Dig-labeled probe specific for the junction site of circSMAD3 was designed and synthesized by Sangon bio. (Shanghai, China). Antibody against Digoxigenin was from Abcam company (ab51949, USA). TSA amplification system was purchased from servicebio company (Wuhan, China). The protocol was performed as manufacturer provided. The signals of the probe were detected and the images were acquired on Lei TCS SP8 Laser Scanning Confocal Microscope (Leica Microsystems, Mannheim, Germany).

**Immunofluorescence analysis**

For cells, HASMC or mASMC were collected and fixed in 4% paraformaldehyde, then permeabilized with 0.5% triton in PBS for 15min and incubated in 5% Bovine Serum Albumin (BSA) for 1hour at room temperature following incubated at 4 °C overnight with primary antibodies diluted in 5% BSA. For carotid arterial tissues or aortic tissues, the pieces were treated by backing, dewaxing, antigen retravel and blocking. Next, the pieces were incubated with primary antibodies diluted in 5% BSA at 4 °C overnight. The primary antibodies were used as followed: anti-hnRNPA1 antibody (CL488-67844, 1:200, Proteintech, China), anti-WDR76 antibody (25528-1-AP, 1:200, Proteintech, China), anti-α-SMA antibody (ABM0052, 1:200, Abbkine, China) and anti-Ki67 antibody (Abclonal, China). After overnight, the samples were incubated with secondary antibodies (FITC mouse, CY3 rabbit, Servicebio, Wuhan, China) at 1:500 for 45min, and washed three times with PBS. DAPI were incubated for another 15min. The pictures were taken on a fluorescent microscope (Axio Imager 2, Zeiss, Germany).

**Isolation of mouse primary aortic smooth muscle cell**

The isolation of primary aortic smooth muscle cells was conducted as the previous protocols. Briefly, adult mice (n=10) were sacrificed with being anesthetized with xylazine (5 mg/kg) and ketamine (80 mg/kg) (Sigma, USA) and were disinfected in 75% ethanol for 5 minutes. Then, the skin was cut and the aortic was isolated. The inner layer and adventitia were discarded. The media layer was cut into pieces, then were transferred to the 15ml tube with 5ml collagenase type 2 (1mg/L, Washonton, USA) and incubated at 37 °C water bath for 1hour. After digestion, the supernatant was neutralized by 1ml serum, filtrated through a cell strainer (200 meshes) and centrifuged at 4 °C and 1000g for 8min. The isolated cells were in the sediment and resuspended using DMEM with 20% fetal bovine serum to be seeded to culture flask and cultured. The verification of mouse primary smooth muscle cells was confirmed by immunoprecipitation analysis with antibody against α-SMA.

**Cell morphology measure**

HASMC and mASMC were seeded on a glass coverslip in 24-well dishes. After stimulation, the cells were washed by PBS buffer, fixes in 4% paraformaldehyde (Servicebio, Wuhan, China) and permeabilized with 0.5% Triton X-100 (Beyotime, Shanghai, China) for 15min respectively. Then, the sample were incubated with 0.1% phalloidin (Abclonal, Wuhan, China) for 30 min at 37 °C, following being incubated with DAPI at 1:100 dilution for another 15min. coverslips were mounted on glass slides with mounting media. The pictures of cell morphology were taken using fluorescent microscopy (Axio Imager 2, Zeiss, Germany). This process was replicated in a least three independent experiments.

**Collagen Gel Contraction Assay**

The collagen gel contraction assay was conducted by using Cell Contraction Assay Kit (CBA-021, Cell Biolabs, San Diego, CA, USA). In brief, after treatment for 24 hours, HASMC and mASMC were resuspended in DMEM containing 10% FBS at a density of 5×10^5^ cells/ml. Collagen Gel Working Solution was prepared according to the protocols and then mixed with the cell suspension in a volume radio of 4:1. After that, 0.5 mL of the cell contraction matrix was added to each well of the 24-well plate followed by incubation at 37 °C for 1 hour. Then, 1.0 ml of DMEM that containing 10% FBS was added atop each collagen gel lattice. 24 hours later, the plate was scanned and the area of gel was analyzed by using ImageJ Pro Plus 6.0 software.

**CCK8 assay**

HASMC and mASMC were seeded into 96-well plates at appropriate number 10000 cells, and infected by circSMAD3 overexpression or shRNA adenovirus. The proliferation of cells was monitored at 48h or 72h by CCK8 kit as the manufacturer’s instructions. The OD value was detected at 450nm wave length by Biotek synergy2 (USA).

**Transwell assay**

This experiment was conducted as previously described. Costar chambers containing transwell inserts with a pore size of 8μm (Corning, NY, USA) were used for this assay. Briefly, after being treated for 24 hours, HASMC or mASMC were added to upper transwell chambers in 200ul DMEM with 10% FBS, while 600ul fresh medium containing 10% FBS was deposited in the bottom chamber, and cultured at 37ºC and 5% CO2 for 24 h. after that, cells at the upper layer were scraped with cotton swabs, and then migrated cells at the down layer were fixed with 4% formaldehyde and incubated with crystal violet solution (0.2%). Cells were imaged and counted under microscope (Axio Imager 2, Zeiss, Germany).

**Wound healing assay**

Wound healing assay was performed as previously mentioned. In brief, HASMC and mASMC were seeded in 6-well plate with appropriate number. After the cells was stimulated for 24 hours, the supernatant was discarded and the cells were washed using 1x PBS buffer. 200ul tips were used to scratch and the floating cells were washed away by 1x PBS buffer. The cells continued to be cultured by DMEM with free FBS at 37 °C. The pictures were taken by microscope (Axio Imager 2, Zeiss, Germany), and the gap was calculated by ImageJ Pro Plus 6.0 software.

**5-Ethynyl-2'-deoxyuridine (EDU) assay**

The EDU assay was conducted according to the Cell-Light^TM^ EdU Apollo In Vitro Kit (RiboBio, Guangzhou, China). HASMC and mASMC were seeded into 48-well plates. After intervention, the cells were incubated with EDU solution at 1:1000 dilution at 37 °C for 2 hours, fixed by 4% paraformaldehyde and incubated with master buffer prepared as the protocols. DAPI was incubated for 15min. The pictures were taken by fluorescence microscope (Axio Imager 2, Zeiss, Germany).

**Flow cytometry assay to detect cell apoptosis**

The level of cell apoptosis was detected by 7-AAD and Annexin V staining using Annexin V-APC/7-AAD Apoptosis Detection Kit (Kaigentec, Jiangsu, China). The HASMCs were cultured in 6-well plates and infected with circSMAD3 overexpression and circSMAD3 sh-RNA adenovirus for 48 hours. After that, the cells were collected and staining with Annexin V-APC/7-AAD at 37℃ for 30 min. and then the samples were detected by flow cytometer (BD Biosciences) and the data were analyzed using FlowJo software (TreeStar).

**RNA sequencing and data analysis**

RNA library and sequencing was conducted by Frasergen Co (Wuhan,China). In brief, the total RNA was extracted from HASMCs infected by circSMAD3 sh-RNA adenovirus for 48 hours. The concentration and purification of RNA was detected by Nanodrop 2000. For the RNA library conduction, the total RNA was processed by rRNA removal method to enrich mRNA. The obtained RNA was fragmented, reverse transcribed to form double-stranded DNA. After being flattened and phosphorylated at the 5 'end and forming A sticky end with an "A" protruding from the 3' end, the resultant double-stranded DNA ends were connected to a bubble-like linker with a "T" protruding from the 3 'end. The ligation products were amplified by PCR with specific primers and thermally denatured to single stranded DNA, and then the single stranded DNA was looped by a bridge primer to generate a single stranded circular DNA library.

For the analysis of RNA sequencing, the primary data was cleaned by Trim_galore, aligned with reference genome (Hg38) by Bowtie2, and then analyzed by RSEM to obtain the FPKM (Fragments Per Kilobase per Million bases) in each group. the differential expression of genes was calculated by Gene Ontology, KEGG and GSEA analysis. The raw data were uploaded into GEO database (accession number: PRJNA1016164).

**Chromatin immunoprecipitation assay (ChIP)**

Chromatin immunoprecipitation assay was conducted to verified the binding intensity of p53 on its target genes (P21, PUMA and NOXA) promotor region with circSMAD3 overexpression or silence according to the ChIP Assay Kit (Biyotime, Wuhan, China). In brief, the cells were crosslinked by 1% Formaldehyde, neutralized by 2mg/ml glycine, then washed by PBS and then lysed by lysis buffer. The cell lysis was sonicated to break DNA into pieces and then centrifuged. The supernatants were incubated with antibody against p53 overnight with shaking. The complex was washed according to the protocols and purified by DNA purification kit (Biyotime, Wuhan, China). Binding intensity of p53 on its target gene promotors was identified by qPCR. The primers of target gene promotor region were listed in **Supplementary Table 4**.

**RNA pulldown assay and Mass Spectrum**

The probe labelled by biotin and specific to circSMAD3 was designed and synthesized by RiboBio (Guangzhou, China). Firstly, circSMAD3 probe was incubated with streptavidin magnetic beads (Life Technologies, USA) at room temperature for 1 hour. Then, cell lysates were incubated with probe-coated beads at room temperature for another 2 hours. The complexes were washed two times and divided to two parts for RNA and protein extraction. the bound RNA in the pulldown materials were extracted using Trizol reagent and analyzed by qRT-PCR assay and PCR. The proteins interacting with circSMAD3 were detected by Mass Spectrum or western blot. Mass Spectrum is conducted by Genecreate Bio (Wuhan, China). The probe sequence used in the study were showed in **Supplementary Table 5**. The proteins identified in HASMC by Mass spectrum were showed in **Supplementary Table 6**. The protein s related with VSMC phenotype in GO database and literatures were showed in **Supplementary Table 7**.

**RNA immunoprecipitation (RIP)**

RIP was conducted as previously. Briefly, the antibody was incubated with protein A/G beads for 1 hours at room temperature. Meanwhile, cells were lysed by IP lysis containing Rnase inhibitor and protease inhibitor and centrifuged to retain the supernatant. After that, the antibody-beads complexes were incubated with cell supernatant overnight at 4 °C. The protein-RNA complex was extracted by Trizol. The enrichment of circSMAD3 and pre-p53 was tested by qRT-PCR. The antibodies against hnRNPA1, WDR76, YBX1, PHB2, PCBP1, SMAD3, SMAD4 and IgG used for RIP as mentioned in western blot.

**Hematoxylin and eosin (H&E) staining**

The pieces of carotid artery were treated by backing, dewaxing, then placed in a metal staining rack, and immersed in the filtered Harris Hematoxylin for 10 seconds. After that, the sections were washed by water until the water wass clear, and then immersed in EOSIN stain for 30 seconds, and continued to be washed by water until the water is clear. And then, the pieces were dehydrated in ascending alcohol solutions (50%,70%,80%,95% x 2, 100% x 2) and cleared with xylene in staining dish. The pictures were taken by fluorescence microscope (Axio Imager 2, Zeiss, Germany).

**Verhoeff Van Gieson (EVG) staining**

The Verhoeff Van Gieson (EVG) staining was performed as the Manufacturer instructions (G1590, Solarbio, Beijing, China). In brief, the pieces of carotid artery were treated by backing and dewaxing. Then, the pieces were put into the Weigert resorcin fuchsin stain solution for incubating 1 hour at room temperature, differentiated in acid alcohol and washed in water 10 minutes. After that, the sections were Stained in Van Gieson for 5 minutes, then washed in water, dehydrated in ethanol with the concentration at 75%, 85%, 95% and 100%, and eventually cleared. The pictures were taken by fluorescence microscope (Axio Imager 2, Zeiss, Germany).

**TUNEL staining**

The TUNEL staining was conducted according to the protocol provided by the Meilun biotechnology Co.Ltd (MA0224, Dalian, China). The pieces of carotid artery were treated by backing and dewaxing as above, then permeabilized with proteinase K (20μg/ml) for incubating 30 minutes at 37℃. After that, the pieces were incubated with TUNEL detecting solution for another 1 hour at 37℃. The pictures were taken by fluorescence microscope (Axio Imager 2, Zeiss, Germany).

**Construction of carotid arterial injury model and virus infection**

C57B/J mice were purchased from Gempharmatech Co., Ltd (Jiangsu, China) aged 7 weeks. Eight-week-old C57BL/6J mice was injected with circSMAD3 overexpression or sh-RNA adeno-associated virus at a titer of 5 × 10^11^ v.g./mL (100μL per mouse) by tail vein. After 3 weeks, the mice were induced to arterial injury model on the right common carotid arterial by wire. Briefly, the mice were anesthetized with 1% pentobarbital (Sigma, USA), and fixed on the plate. The carotid skin and connective tissue were cut off and separated to clearly expose the common, internal and external carotid arterial under a dissecting microscope. Then the proximal common carotid arterial and internal carotid arterial were clipped for the temporary blockage of blood flow and the distal external carotid arterial was ligated with 7-0 silk sutures. Then, a wire (0.38 mm) was introduced from external carotid arterial into the common arterial lumen and withdrawn 5 times. After that, the proximal distal carotid arterial was ligated to seal the breach. The proximal common carotid arterial and internal carotid arterial were subsequently released to restore blood flow. The operated mice were kept warm and revived on an electric blanket. After two weeks, the carotid arteries were collected. Some samples were embedded in wax for histological studies, and the others were frozen to be used for RNA and protein extraction experiments. Neointimal thickening was visualized by hematoxylin and eosin (H&E) staining and analyzed by determining the ratio of intima to media thickness. Cell proliferation was evaluated by immunofluorescence staining using rabbit anti-Ki67 primary antibody (28074-1-AP, Proteintech, China) and goat antirabbit cy3 (Servicebio, Wuhan, China) secondary antibody. Phenotypic switching was assessed by measuring the expressions of α-SMA and then quantified by Western blotting of injured C57BL/6J carotid arteries.

**Ethics Statement and human aortic tissue acquisition**

All mice were maintained and studied using protocols in accordance with the NIH Guide for the Care and Use of Laboratory Animals and approved by the Committee on the Ethics of Animal Experiments of the Animal Research Committee of Tongji Medical College, Huazhong University of Science and Technology. ([2022] IACUC Number3836).

**Statistical analysis**

Statistical analysis was conducted using SPSS22.0 (IBM, SPSS, Chicago, IL, USA) and GraphPad Prism8. Student's *t*-test, one-way or two-way analysis of variance (ANOVA) was applied in multiple different groups experiment statistical analysis. All data are presented as the Mean ± Standard deviation (SD) or mean ± Mean and Standard error of Mean (SEM) from three independent experiments. p-value <0.05 was considered statistically significant.

## Supplemental Tables

**Supplemental Table 1.** List of primers used in qPCR.

| Primer name | Forward | Reverse |
| --- | --- | --- |
| h-circSMAD3 | CAGCACATAATAACTTGGGTCCCT | AACTCACACAGCTCCATGGC |
| h-ACTA2 | CTATGCCTCTGGACGCACAACT | CAGATCCAGACGCATGATGGCA |
| h-CNN1 | CCAACGACCTGTTTGAGAACACC | ATTTCCGCTCCTGCTTCTCTGC |
| h-TAGLN | TCCAGGTCTGGCTGAAGAATGG | CTGCTCCATCTGCTTGAAGACC |
| h-hnRNPA1 | GCCCTGTCAAAGCAAGAGATGG | CTTCATTGCCGTCCTCTCTAGG |
| h-SMAD3 | CAGAGAGTAGAGACACCAGTTC | ATGCTGTGGTTCATCTGGTGGT |
| h-GAPDH | AGAAGGCTGGGGCTCATTTG | AGGGGCCATCCACAGTCTTC |
| h-p53 | CAGCCAAGTCTGTGACTTGCA | GTGTGGAATCAACCCACAGCT |
| h-P53β | GAGCACTAAGCGAGCACTGCC | TTGAAAGCTGGTCTGGTCCTGA |
| h-△133p53 | ACTCTGTCTCCTTCCTCTTCCTACAG | GTGTGGAATCAACCCACAGCT |
| h-△40p53 | TGAGTGGATCCATTGGAAGG | GTCTGAAAGACAAGAGCAGAAAG |
| h-p53γ | AACCACTGGATGGAGAATATTTCAC | TCAACTTACGACGAGTTTATCAGGAA |
| h-TSp53 | TCACCATCATCACACTGGAAGAC | CACGCACCTCAAAGCTGTTC |
| h-pre-p53 | CACCTTTCCTTGCCTCTTTCC | CCACTTGATAAGAGGTCCCAAGAC |
| h-GADD45G | CGTCTACGAGTCAGCCAAAGTC | CGATGTCGTTCTCGCAGCAGAA |
| h-SESN3 | GACAGTGACCTGCTATCCTGAG | CCGAGTTATGGCACGAAGAGCA |
| h-FAS | GGACCCAGAATACCAAGTGCAG | GTTGCTGGTGAGTGTGCATTCC |
| h-GADD45A | CTGGAGGAAGTGCTCAGCAAAG | AGAGCCACATCTCTGTCGTCGT |
| h-THBS1 | GCTGGAAATGTGGTGCTTGTCC | CTCCATTGTGGTTGAAGCAGGC |
| h-CASP3 | GGAAGCGAATCAATGGACTCTGG | GCATCGACATCTGTACCAGACC |
| h-DDB2 | CCAGTTTTACGCCTCCTCAATGG | GGCTACTAGCAGACACATCCAG |
| h-PERP | TGAGCGAGTTGGTCTACACGGA | ACCATCACGCTGATGCTTGGCT |
| h-ZMAT3 | GCTCTGTGATGCCTCCTTCAGT | TTGACCCAGCTCTGAGGATTCC |
| h-CCNE1 | TGTGTCCTGGATGTTGACTGCC | CTCTATGTCGCACCACTGATACC |
| h-RRM2-S | CTGGCTCAAGAAACGAGGACTG | CTCTCCTCCGATGGTTTGTGTAC |
| h-CASP8-S | AGAAGAGGGTCATCCTGGGAGA | TCAGGACTTCCTTCAAGGCTGC |
| h-SESN2 | AGATGGAGAGCCGCTTTGAGCT | CCGAGTGAAGTCCTCATATCCG |
| h-IGFBP3 | CGCTACAAAGTTGACTACGAGTC | GTCTTCCATTTCTCTACGGCAGG |
| h-CCNB2 | CAACCAGAGCAGCACAAGTAGC | GGAGCCAACTTTTCCATCTGTAC |
| h-ADGRB1 | ACCTGTTGGCAGAGGAGAATCG | GGTTGTCTGTCACCTGGTATGC |
| h-CCND1 | TCTACACCGACAACTCCATCCG | TCTGGCATTTTGGAGAGGAAGTG |
| h-CDK2 | ATGGATGCCTCTGCTCTCACTG | CCCGATGAGAATGGCAGAAAGC |
| h-SERPINE1 | CTCATCAGCCACTGGAAAGGCA | GACTCGTGAAGTCAGCCTGAAAC |
| h-GTSE1 | CTCTACCAGCAATCTCGCAAGG | GACTTGCTGATGTTTGACAGAGG |
| h-CCNB1 | GACCTGTGTCAGGCTTTCTCTG | GGTATTTTGGTCTGACTGCTTGC |
| h-CDK1 | GGAAACCAGGAAGCCTAGCATC | GGATGATTCAGTGCCATTTTGCC |
| h-APAF1 | GCCAAGCAGGAGGTCGATAATG | GACCATCCTCAGAAAAGCAGGC |
| h-CCNE2 | CTTACGTCACTGATGGTGCTTGC | CTTGGAGAAAGAGATTTAGCCAGG |
| h-SFN | TGCTGGACAGCCACCTCATCAA | GGCTGAGTCAATGATGCGCTTC |
| m-circSMAD3 | CAGCACACAATAACTTGGGTCACT | AACTCACAGAGCTCCATGGC |
| m-ACTA2 | TGCTGACAGAGGCACCACTGAA | CAGTTGTACGTCCAGAGGCATAG |
| m-CNN1 | ACAAGAGCGGAGATTTGAGCCG | TCATAGAGGTGACGCCGTGTAC |
| m-TAGLN | GCAGATGGAACAGGTGGCTCAA | CCCAAAGCCATTAGAGTCCTCTG |
| m-GAPDH | CATCACTGCCACCCAGAAGACTG | ATGCCAGTGAGCTTCCCGTTCAG |
| mmu_circ_0015010 | GCAACTACCACCACGGCTTT | ACTCGCTGTGCCTCGGAAC |
| mmu_circ_0007526 | AAGGGCTGGGGAGCAGAATA | GAAGACGGCCATCAAGAGACAT |
| mmu_circ_0007524 | GCCTTCAGAGTGGACCTTTTG | GCTGGTTAGTATTCCTGTGTTTCA |
| mmu_circ_0007528 | AGAGATACGGCTGGCACCC | GAGAGTAGTAGGAGACAGTTCAGCC |
| mmu_circ_0007525 | ATCGGAGGCAGACAGTAACAAGT | CAGTCCCCAAATTTCAGAGCA |
| cicRNA.758 | CCCTTCAGTGCGATGCTCA | GTCTCTTCACCACTGGCGGA |
| cicRNA.757 | GAATGAGTTTTGTGAAGGGCTG | CCAAAGGTAACAATCCACACTCA |
| cicRNA.756 | ATGACTACACCCACTCCATTCCA | GGTGTGAAACCTGAAGACGGC |
| mmu_circ_0007523 | ATCGGAGGCAGACAGTAACAAGT | GGCAAGATGGACGACATTTTC |
| cicRNA.755 | CGTAAACCCATCAAAGACTCG | GCCTGTTGTGTCCCACTGAT |
| mmu_circ_0007527 | TACCTATGCCAACAGCAGCCT | CTGGGACTCCTCCATTCTCACT |
| cicRNA.759 | CTTGCCTCCAGTCTTAGTGCCT | CCAAAGGTAACAATCCACACTCAG |
| cicRNA.19174 | CTGTCTACCAGTTGACCCGAATG | GCAGCGAACTCCTGGTTGTT |
| cicRNA.19175 | CAGCACACAATAACTTGGGTCACT | AACTCACAGAGCTCCATGGC |
| mmu_circ_0015778 | TTCCATCCCCGAAAACACTAACTT | AGTGGTAGGGATTCACGCAGAC |
| mmu_circ_0007495 | AGCCAGGACAGCAGCAGAAT | AGGCATCGTTACTTGTTGGTGT |
| cicRNA.812 | GCCCCATCCTGGACATTAC | TTCACTAACATACTTGGAGCAGG |
| mmu_circ_0007497 | ATACAGAGAACATTGGATGGACG | TGCTAGGATGAGCTCCATTTGT |
| mmu_circ_0007492 | TCCACAGGACAGAAGCGATTG | CAATGGCCAATGTTGCAAGA |
| mmu_circ_0007493 | TTGCCTCACCACCAAAACG | GCAACAGTCCTTCACTATGGCT |
| mmu_circ_0000894 | CTATGCCCGTCTGTGGAGGT | CAGGCATCGTTACTTGTTGGTG |
| mmu_circ_0007496 | AGCCGTCCTTACCCACTGAA | ATCTGGTGAGCAGGATGACTTG |
| mmu_circ_0004681 | CAACGCTCCTTTCCCCTTATC | GAGGCGGGGAGTGACATTTA |
| mmu_circ_0004682 | CTTCCATCCCACCACCGTC | CAGGTGCCATAGGCTTCTTCTTA |
| cicRNA.8390 | GGATGGACGTCTGCAAGTTTC | GGTGCCATAGGCTTCTTCTTAGT |

**Supplemental Table 2.** List of primers used in PCR.

| Primer name | Forward | Reverse |
| --- | --- | --- |
| hcircSMAD3 | CCTGGCTACCTGAGTGAAGATG | GCACCAACACAGGAGGTAGAAC |
| mcircSMAD3 | TCTCCAAACCTCTCCCCGAAT | CCACAGGCGGCAGTAGATAAC |

**Supplemental Table 3.** List of siRNAs in this study.

| siRNA ID | sequence |
| --- | --- |
| si-h-circSMAD3 | CATAATAACTTGGGTCCCTGG |
| si-h-hnNRPA1 | AGATATTTGTTGGTGGCATTA |
| Si-p53γ-3 | TTCGATGGTGTTACTTCCTGATA |

**Supplemental Table 4.** List of primers used in ChIP-qPCR.

| Primer name | Forward | Reverse |
| --- | --- | --- |
| h-p21-chip-1 | GGACATTTGACAACCAGCCC | CTCCCCTGGACTTCACCTTTG |
| h-p21-chip-2 | AGGTCAGGGGTGTGAGGTAGAT | AGAGACTACCAAAAAAGGGCAAC |
| h-p21-chip-3 | ATCAGGTTGCCCTTTTTTGGTA | CCACTCTGGCAGGCAAGG |
| h-p21-chip-4 | TGGGTAAATCCTTGCCTGC | CGCGGCCCTGATATACAAC |
| h-PUMA-chip-1 | GGAAGGGACAATGAATAATCGG | TTCTCATTGTTACTTCCTGCCCT |
| h-PUMA-chip-2 | GGGCAGGAAGTAACAATGAGAAA | GCCAAGGTGCGTGGACTG |
| h-PUMA-chip-3 | CGGAGTTTGCTCTTGTTGCC | TCACAAACAACCCTACCGAACA |
| h-PUMA-chip-4 | GTGCCTGTTCGGTAGGGTTG | ACCCCCAGCGATGCGTAC |
| h-NOXA-chip-1 | GGGATAATAGATTTCAAGGCAGC | GCCACACCCTTTAGAGGAACC |
| h-NOXA-chip-2 | AGGTTCCTCTAAAGGGTGTGGC | CCTGTCGTCCCAGTTGCTTG |
| h-NOXA-chip-3 | CCCAGGTTCAAGCGATTCTC | GCACTGCCCTGATTCCGA |
| h-NOXA-chip-4 | TGGTTCGGAATCAGGGCA | ACTTGAAGAGTATGGGCAGTAACAG |
| h-NOXA-chip-5 | TCCCTGTTACTGCCCATACTCTT | GGCGTTATGGGAGCGGAC |
| h-NOXA-chip-6 | GTCCGCTCCCATAACGCC | AATACTTTGGCTTGATAATGAACACT |

**Supplemental Table 5.** List of probs for RNA pulldown.

| Prob ID | sequence |
| --- | --- |
| h-circSMAD3 prob | TGTCCCCAGCACATAATAACTTGGGTCCCTGGATGGCCGG |
| m-circSMAD3 prob | TGTCCCCAGCACACAATAACTTGGGTCACTGGATGGTCGGC |

**Supplemental Table 6.** Proteins identified by mass spectrum in HASMC.

| **Gene** | **Mw(kDa)** | **Length** | **Score** | **Unique peptides** |
| --- | --- | --- | --- | --- |
| IQGAP1 | 189.252 | 1657 | 240.98 | 33 |
| ACTB | 41.737 | 375 | 226.35 | 1 |
| VIM | 53.652 | 466 | 195.16 | 23 |
| HNRNPM | 77.516 | 730 | 132.41 | 14 |
| YBX1 | 35.924 | 324 | 125.14 | 11 |
| HNRNPU | 90.584 | 825 | 111.62 | 15 |
| MYH9 | 226.532 | 1960 | 111.59 | 16 |
| MAP4 | 121.005 | 1152 | 102.55 | 14 |
| CKAP4 | 66.022 | 602 | 93.324 | 13 |
| ATP5F1A | 59.751 | 553 | 80.694 | 10 |
| HDLBP | 141.456 | 1268 | 69.615 | 7 |
| PARP1 | 113.084 | 1014 | 65.879 | 10 |
| COPA | 138.346 | 1224 | 64.675 | 10 |
| RPS3A | 29.945 | 264 | 58.012 | 7 |
| RPL13 | 24.261 | 211 | 56.469 | 6 |
| TUBB4B | 49.831 | 445 | 53.899 | 2 |
| PKM | 57.937 | 531 | 52.013 | 7 |
| RPS19 | 16.061 | 145 | 51.634 | 6 |
| TUBA1C | 49.895 | 449 | 51.564 | 6 |
| HNRNPK | 50.976 | 463 | 51.338 | 7 |
| CAVIN1 | 43.476 | 390 | 51.162 | 7 |
| HNRNPA1 | 38.747 | 372 | 50.02 | 3 |
| SFPQ | 76.149 | 707 | 48.777 | 6 |
| LASP1 | 29.717 | 261 | 46.354 | 4 |
| HNRNPAB | 36.225 | 332 | 44.319 | 1 |
| ATAD3A | 71.369 | 634 | 43.894 | 7 |
| RPL7 | 29.226 | 248 | 42.773 | 4 |
| NCL | 76.614 | 710 | 41.273 | 6 |
| RPL5 | 34.363 | 297 | 40.829 | 5 |
| RPN1 | 68.569 | 607 | 40.085 | 5 |
| MYO1C | 121.682 | 1063 | 39.715 | 6 |
| NONO | 54.232 | 471 | 39.425 | 6 |
| HADHA | 83 | 763 | 37.873 | 4 |
| CALD1 | 93.231 | 793 | 36.492 | 5 |
| H1-4 | 21.865 | 219 | 32.415 | 4 |
| RPL8 | 28.025 | 257 | 31.925 | 4 |
| RPL26 | 17.258 | 145 | 31.659 | 5 |
| ARHGEF2 | 111.543 | 986 | 31.407 | 5 |
| EEF1A1 | 50.141 | 462 | 29.986 | 4 |
| RPS14 | 16.273 | 151 | 28.246 | 3 |
| PTBP1 | 57.221 | 531 | 27.685 | 4 |
| HP1BP3 | 61.207 | 553 | 27.681 | 4 |
| PCBP1 | 37.498 | 356 | 27.668 | 3 |
| HSPA5 | 72.333 | 654 | 27.446 | 2 |
| RPL11 | 20.252 | 178 | 27.318 | 4 |
| ATP5PO | 23.277 | 213 | 27.316 | 4 |
| RPS18 | 17.719 | 152 | 27.064 | 4 |
| SERBP1 | 44.965 | 408 | 26.852 | 2 |
| HSPA8 | 70.898 | 646 | 26.693 | 4 |
| VARS1 | 140.476 | 1264 | 26.353 | 3 |
| RPL9 | 21.863 | 192 | 25.494 | 4 |
| UBA52 | 14.728 | 128 | 25.218 | 3 |
| SLC25A6 | 32.866 | 298 | 24.627 | 4 |
| RPL13AP3 | 12.135 | 102 | 24.302 | 4 |
| RPS11 | 18.431 | 158 | 24.295 | 3 |
| EPRS1 | 170.591 | 1512 | 24.282 | 4 |
| RPL19 | 23.466 | 196 | 24.085 | 3 |
| HMGB2 | 24.034 | 209 | 23.932 | 2 |
| HMGA2 | 11.832 | 109 | 23.66 | 1 |
| RPL31 | 14.463 | 125 | 23.518 | 4 |
| RPS4X | 29.598 | 263 | 23.302 | 3 |
| KHDRBS1 | 48.227 | 443 | 22.328 | 3 |
| H1-5 | 22.58 | 226 | 21.462 | 3 |
| RPS10 | 18.898 | 165 | 21.01 | 3 |
| PDLIM7 | 49.845 | 457 | 20.902 | 3 |
| CPT1A | 88.368 | 773 | 20.85 | 3 |
| RPL24 | 17.779 | 157 | 20.827 | 3 |
| RPL29 | 17.752 | 159 | 20.058 | 2 |
| G3BP2 | 54.121 | 482 | 19.822 | 3 |
| HADHB | 51.294 | 474 | 19.791 | 3 |
| ANXA2 | 38.604 | 339 | 19.715 | 3 |
| ATP5F1B | 56.56 | 529 | 19.65 | 3 |
| XRCC6 | 69.843 | 609 | 19.325 | 3 |
| RPS20 | 13.373 | 119 | 19.31 | 3 |
| RPL23A | 17.695 | 156 | 19.186 | 2 |
| RALA | 23.567 | 206 | 19.042 | 2 |
| RPS5 | 22.876 | 204 | 18.987 | 2 |
| FARSA | 57.564 | 508 | 18.986 | 3 |
| HMGN2 | 9.393 | 90 | 18.689 | 1 |
| FAM120A | 121.888 | 1118 | 18.383 | 3 |
| ABCF2 | 71.29 | 623 | 18.212 | 3 |
| RPL21 | 18.565 | 160 | 17.925 | 2 |
| HNRNPA3 | 39.595 | 378 | 17.707 | 2 |
| HNRNPA2B1 | 37.43 | 353 | 17.542 | 3 |
| IFI16 | 88.256 | 785 | 15.573 | 2 |
| TPM1 | 32.709 | 284 | 14.944 | 0 |
| RPS13 | 17.222 | 151 | 14.787 | 1 |
| TUBB | 49.671 | 444 | 14.632 | 0 |
| GTF2I | 112.416 | 998 | 14.45 | 2 |
| MTDH | 63.837 | 582 | 13.931 | 2 |
| PPP1R12A | 115.281 | 1030 | 13.792 | 1 |
| GAPDH | 36.053 | 335 | 13.79 | 2 |
| FKBP11 | 22.18 | 201 | 13.743 | 2 |
| RPL22 | 14.787 | 128 | 13.552 | 2 |
| DDX5 | 69.148 | 614 | 13.41 | 1 |
| XRN2 | 108.582 | 950 | 13.182 | 2 |
| PRRC2A | 228.863 | 2157 | 13.157 | 2 |
| PRRC2C | 316.911 | 2896 | 13.008 | 2 |
| HNRNPD | 38.434 | 355 | 12.974 | 2 |
| FAU | 14.39 | 133 | 12.829 | 2 |
| ALYREF | 26.888 | 257 | 12.593 | 2 |
| KRAS | 21.656 | 189 | 12.524 | 2 |
| HMGA1 | 11.676 | 107 | 12.458 | 2 |
| ATP5ME | 7.933 | 69 | 12.432 | 2 |
| SLC25A3 | 40.095 | 362 | 12.382 | 1 |
| RAN | 24.423 | 216 | 12.352 | 2 |
| SSBP1 | 17.26 | 148 | 12.329 | 2 |
| RPS15 | 17.04 | 145 | 12.326 | 2 |
| DNAJC1 | 63.883 | 554 | 12.311 | 2 |
| EHD2 | 61.161 | 543 | 12.282 | 1 |
| SND1 | 101.997 | 910 | 12.262 | 2 |
| RAC1 | 21.45 | 192 | 12.183 | 2 |
| WDR76 | 62.553 | 562 | 12.101 | 2 |
| CNTRL | 268.886 | 2325 | 12.055 | 2 |
| RBMX | 42.332 | 391 | 11.986 | 2 |
| RPL17 | 21.397 | 184 | 11.916 | 2 |
| RPS7 | 22.127 | 194 | 11.874 | 2 |
| RPS6 | 28.681 | 249 | 11.849 | 2 |
| PURA | 34.911 | 322 | 11.741 | 2 |
| ASPH | 85.863 | 758 | 11.739 | 2 |
| KRT6B | 60.067 | 564 | 11.662 | 0 |
| SHMT2 | 55.993 | 504 | 11.577 | 2 |
| TMEM214 | 77.151 | 689 | 11.541 | 2 |
| DCD | 11.284 | 110 | 11.493 | 2 |
| CCDC124 | 25.835 | 223 | 11.476 | 2 |
| TMPO | 50.67 | 454 | 11.464 | 2 |
| ABHD12 | 45.097 | 398 | 11.444 | 2 |
| RPL3 | 46.109 | 403 | 10.705 | 1 |
| ACTG1 | 41.793 | 375 | 9.6985 | 1 |
| EIF2S3B | 51.229 | 472 | 9.0423 | 1 |
| HNRNPA0 | 30.841 | 305 | 8.9086 | 1 |
| HSPB1 | 22.783 | 205 | 8.845 | 1 |
| SLC25A11 | 34.062 | 314 | 8.2766 | 1 |
| NUP153 | 153.938 | 1475 | 8.2604 | 1 |
| CAVIN3 | 27.701 | 261 | 8.0665 | 1 |
| HMGB1 | 24.894 | 215 | 7.9984 | 1 |
| EPB41L3 | 120.678 | 1087 | 7.8134 | 1 |
| HNRNPUL2 | 85.105 | 747 | 7.662 | 1 |
| ZC3H15 | 48.602 | 426 | 7.656 | 1 |
| MTHFD1 | 101.531 | 935 | 7.6355 | 1 |
| SRPRA | 69.811 | 638 | 7.5949 | 1 |
| SYNCRIP | 69.603 | 623 | 7.531 | 1 |
| RAB7A | 23.49 | 207 | 7.4735 | 1 |
| LMNA | 74.139 | 664 | 7.4299 | 1 |
| SLC25A24 | 53.354 | 477 | 7.1895 | 1 |
| UACA | 162.505 | 1416 | 7.1054 | 1 |
| LNPK | 47.74 | 428 | 7.0952 | 1 |
| PRSS3 | 32.529 | 304 | 7.0789 | 1 |
| ACSL3 | 80.42 | 720 | 7.0463 | 1 |
| RTCB | 55.21 | 505 | 7.0319 | 1 |
| SEC61B | 9.974 | 96 | 6.9607 | 1 |
| PCYT1A | 41.731 | 367 | 6.901 | 1 |
| EMD | 28.994 | 254 | 6.8834 | 1 |
| TUBB6 | 49.857 | 446 | 6.8555 | 1 |
| RAB1B | 22.171 | 201 | 6.8524 | 1 |
| EIF2S1 | 36.112 | 315 | 6.8177 | 1 |
| RPS29 | 6.677 | 56 | 6.808 | 1 |
| NPM1 | 32.575 | 294 | 6.8002 | 1 |
| SRP72 | 74.606 | 671 | 6.7543 | 1 |
| RECQL | 73.457 | 649 | 6.7081 | 1 |
| GCC1 | 87.811 | 775 | 6.644 | 1 |
| HNRNPC | 33.67 | 306 | 6.6336 | 1 |
| ILK | 51.419 | 452 | 6.5607 | 1 |
| DARS1 | 57.136 | 501 | 6.554 | 1 |
| RPS16 | 16.445 | 146 | 6.5357 | 1 |
| EIF4A1 | 46.154 | 406 | 6.5304 | 1 |
| SUN2 | 80.311 | 717 | 6.5158 | 1 |
| DRG1 | 40.542 | 367 | 6.5138 | 1 |
| MAGT1 | 38.037 | 335 | 6.4937 | 1 |
| CEP170 | 175.293 | 1584 | 6.4562 | 1 |
| DDX3Y | 73.154 | 660 | 6.4427 | 1 |
| BCL2L14 | 36.598 | 327 | 6.4332 | 1 |
| EIF4G2 | 102.362 | 907 | 6.4196 | 1 |
| HTRA2 | 48.841 | 458 | 6.3913 | 1 |
| RHBDF1 | 97.401 | 855 | 6.3512 | 1 |
| RPS17 | 15.55 | 135 | 6.3268 | 1 |
| MYO1D | 116.202 | 1006 | 6.2741 | 1 |
| FLNA | 280.739 | 2647 | 6.2464 | 1 |
| DEK | 42.674 | 375 | 6.2362 | 1 |
| PROC | 52.071 | 461 | 6.2338 | 1 |
| RBM25 | 100.186 | 843 | 6.2287 | 1 |
| SURF4 | 30.394 | 269 | 6.2175 | 1 |
| RPL32 | 15.86 | 135 | 6.1318 | 1 |
| SEC61A2 | 52.248 | 476 | 6.0709 | 1 |
| PCDH15 | 216.069 | 1955 | 6.0483 | 1 |
| UFL1 | 89.595 | 794 | 6.0396 | 1 |
| ELAVL1 | 36.092 | 326 | 6.0175 | 1 |
| CAPRIN1 | 78.366 | 709 | 6.0055 | 1 |
| SRP68 | 70.73 | 627 | 5.9497 | 1 |
| ATP2B3 | 134.197 | 1220 | 5.9412 | 1 |
| KLC1 | 65.31 | 573 | 5.9375 | 1 |
| PEX16 | 38.629 | 336 | 5.9334 | 1 |
| RRBP1 | 152.456 | 1410 | 5.9197 | 1 |
| H2BC13 | 13.952 | 126 | 5.9187 | 1 |
| TRAM1 | 43.072 | 374 | 5.8747 | 1 |
| RPS24 | 15.423 | 133 | 5.8739 | 1 |
| PUF60 | 59.875 | 559 | 5.8585 | 1 |
| SART1 | 90.255 | 800 | 5.851 | 1 |
| RPL12 | 17.819 | 165 | 5.8486 | 1 |
| CASP14 | 27.68 | 242 | 5.8328 | 1 |
| RPL23 | 14.865 | 140 | 5.8283 | 1 |
| RHOA | 21.768 | 193 | 5.8276 | 1 |
| TNIP2 | 48.7 | 429 | 5.8244 | 1 |
| EHD4 | 61.175 | 541 | 5.8228 | 1 |
| H4C1 | 11.367 | 103 | 5.8208 | 1 |
| DIDO1 | 243.873 | 2240 | 5.8177 | 1 |
| RALB | 23.409 | 206 | 5.8132 | 1 |
| RBMS1 | 44.505 | 406 | 5.8132 | 1 |
| TUFM | 49.542 | 452 | 5.8026 | 1 |
| RPS3 | 26.688 | 243 | 5.7985 | 1 |
| RAP1A | 20.987 | 184 | 5.7918 | 1 |
| SH3KBP1 | 73.126 | 665 | 5.7918 | 1 |
| DDOST | 50.801 | 456 | 5.786 | 1 |
| ACSL4 | 79.188 | 711 | 5.7742 | 1 |
| QARS1 | 87.799 | 775 | 5.7697 | 1 |
| TCP1 | 60.344 | 556 | 5.7696 | 1 |
| RPL4 | 47.697 | 427 | 5.746 | 1 |
| SEC62 | 45.862 | 399 | 5.7444 | 1 |
| AHNAK | 629.101 | 5890 | 5.7423 | 1 |
| PRPF3 | 77.529 | 683 | 5.7417 | 1 |
| ACSL1 | 77.943 | 698 | 5.7407 | 1 |
| CDH15 | 88.916 | 814 | 5.7403 | 1 |
| RPS25 | 13.742 | 125 | 5.7169 | 1 |
| IGF2BP3 | 63.705 | 579 | 5.6951 | 1 |
| NDUFS1 | 79.468 | 727 | 5.695 | 1 |
| MAPK10 | 52.585 | 464 | 5.6932 | 1 |

**Supplemental Table 7.** Proteins related with VSMC phenotype in GO database and literatures.

| protein | protein | protein | protein | protein | protein |
| --- | --- | --- | --- | --- | --- |
| ACVR1 | CRIP2 | IL17F | PBLD | SOX9 | PRELP |
| ACVR1B | CTNNB1 | IL2RA | PDE2A | SP1 | PRTN3 |
| ACVR1C | CUL1 | IL6ST | PDGFA | SRF | PSAP |
| ACVR2A | CX3CL1 | ING2 | PDGFB | SSRP1 | TGFBI |
| ACVR2B | CX3CR1 | INHBC | PDGFRB | STAR | VCAN |
| ACVRL1 | CYP11A1 | ITGA8 | PDPK1 | STAT3 | VTN |
| ADAM10 | DAB2 | ITGAV | PEG10 | STK11 | PKM2 |
| ADAM17 | DLK1 | ITGB1 | PIAS1 | STK16 | PCBP1 |
| ADAM9 | DLX1 | ITGB1BP1 | PIK3CA | STRAP | YBX1 |
| AGT | DLX2 | JAG1 | PIK3CB | STUB1 | HNRNPA1 |
| AMH | DTX1 | JAG2 | PIK3CD | TBL1X | PHB2 |
| AMHR2 | DUSP22 | JUN | PIK3CG | TCF12 | ABI3BP |
| ANKRD1 | E2F1 | JUNB | PIN1 | TCF4 | AEBP1 |
| APP | E2F3 | KAT2B | PITX2 | TDGF1 | AGRN |
| ARG1 | E2F4 | KIT | PKN1 | TFDP1 | APOA1 |
| ARHGEF18 | E2F5 | KLF10 | PKN2 | TFDP2 | APOD |
| ARRB1 | EDNRB | KLF5 | PMEPA1 | TGFB1 | APOE |
| ARRB2 | EFEMP2 | LAMA1 | PML | TGFB1I1 | APOH |
| ASCL1 | EGR1 | LEMD3 | PPM1A | TGFB2 | CFP |
| ATP6AP2 | EID2 | LFNG | PPP1CA | TGFB3 | CMA1 |
| AVP | ELK1 | LIMS1 | PPP1CB | TGFBR1 | COL12A1 |
| AXIN1 | ELN | LMOD1 | PPP1CC | TGFBR2 | COL14A1 |
| B4GALT1 | ENG | LPXN | PPP1R15A | TGFBR3 | COL18A1 |
| BAMBI | EP300 | LTBP1 | PRDM16 | TGFBRAP1 | COL5A1 |
| BARX2 | EPC1 | LTBP3 | PRKAA1 | TGIF1 | COL6A1 |
| BCL9 | EREG | LTBP4 | PRKAA2 | TGIF2 | COL6A2 |
| BCL9L | EYA1 | MAGEA1 | PRKAB1 | THBS1 | COL6A3 |
| BMP2 | F11R | MAML1 | PRKCZ | TLE1 | COL8A1 |
| BMP4 | FCER2 | MAP3K1 | PRRX1 | TLE2 | COL8A2 |
| BMP7 | FERMT2 | MAP3K7 | PRRX2 | TLE3 | CP |
| BMPR1A | FGF10 | MAPK1 | PSEN1 | TLE4 | CPXM2 |
| BMPR1B | FGFR2 | MAPK14 | PSEN2 | TLR3 | CTSD |
| BMPR2 | FHL2 | MAPK7 | PTGS2 | TP53 | DEFA1 |
| CACNA1C | FKBP1A | MDK | PTK2 | TP63 | DPT |
| CAV2 | FMOD | MECOM | PTPRK | TRIM33 | EFEMP1 |
| CAV3 | FNTA | MED28 | RBL1 | UBA52 | EMILIN1 |
| CBL | FOS | MEF2A | RBPJ | UBB | FBLN5 |
| CCL2 | FOXC1 | MEF2C | RBX1 | UBC | HAPLN1 |
| CCNC | FOXH1 | MEN1 | RCAN1 | UBE2D1 | HDGF |
| CCND1 | FOXO3 | MEOX2 | RHOA | UBE2D3 | IGFBP7 |
| CCNT1 | FOXO4 | MFNG | RNF111 | UBE2M | LAMA4 |
| CCNT2 | FOXP3 | MKL1 | RPS27A | UBR5 | LAMA5 |
| CD24 | FOXS1 | MKL2 | SERPINE1 | UCHL5 | LAMC1 |
| CD34 | FSHB | MSTN | SERPINF2 | USP9X | LGALS1 |
| CD36 | FURIN | MTMR4 | SFRP1 | USP9Y | LGALS3 |
| CD46 | GATA2 | MYC | SHH | WFIKKN2 | LGALS3BP |
| CDC7 | GATA6 | MYOCD | SIRT1 | WNT1 | LOXL1 |
| CDK8 | GDF15 | NCOR1 | SIX1 | WNT2 | LUM |
| CDK9 | GDF5 | NCOR2 | SKI | WNT4 | MFAP4 |
| CDKN1B | GDF9 | NEDD4L | SKIL | WNT5A | MGP |
| CDKN1C | GIPC1 | NEDD8 | SKP1 | WNT7A | NID1 |
| CDKN2B | GLG1 | NFATC3 | SMAD1 | WNT7B | PCOLCE |
| CGN | GMDS | NFATC4 | SMAD2 | WWOX | SERPINA4 |
| CIDEA | GZMB | NFKBIA | SMAD3 | WWTR1 | SERPINB1 |
| CITED1 | HDAC1 | NKX2-1 | SMAD4 | XCL1 | SERPINF1 |
| CITED2 | HDAC3 | NKX2-5 | SMAD5 | XPO1 | SLPI |
| CLEC3B | HDAC9 | NKX3-1 | SMAD6 | ZEB1 | SOD3 |
| CNTN1 | HES1 | NLK | SMAD7 | ZFYVE9 | SPON1 |
| COL1A1 | HEY1 | NOTCH1 | SMAD9 | APCS | TIMP3 |
| COL1A2 | HEY2 | NOTCH2 | SMARCA2 | BGN | TINAGL1 |
| COL3A1 | HIF1A | NOTCH3 | SMARCA4 | CALR | TNC |
| COL4A1 | HIPK2 | NOTCH4 | SMURF1 | CLU | TNXB |
| COL4A2 | HMGA1 | NOX4 | SMURF2 | CTSG | TPSAB1 |
| COL4A3 | HSPA5 | NPNT | SNAI1 | DCN |  |
| COL4A4 | HSPG2 | NTF3 | SNW1 | FBLN1 |  |
| COL4A5 | HTRA1 | NUMB | SNX6 | FBN1 |  |
| COMP | HYAL2 | ONECUT1 | SOX11 | FN1 |  |
| CREB1 | ID1 | PARD3 | SOX2 | MATN2 |  |
| CREBBP | IGF1 | PARD6A | SOX5 | MMP2 |  |
| CRIP1 | IL13 | PARP1 | SOX6 | NID2 |  |

## Supplement Figure


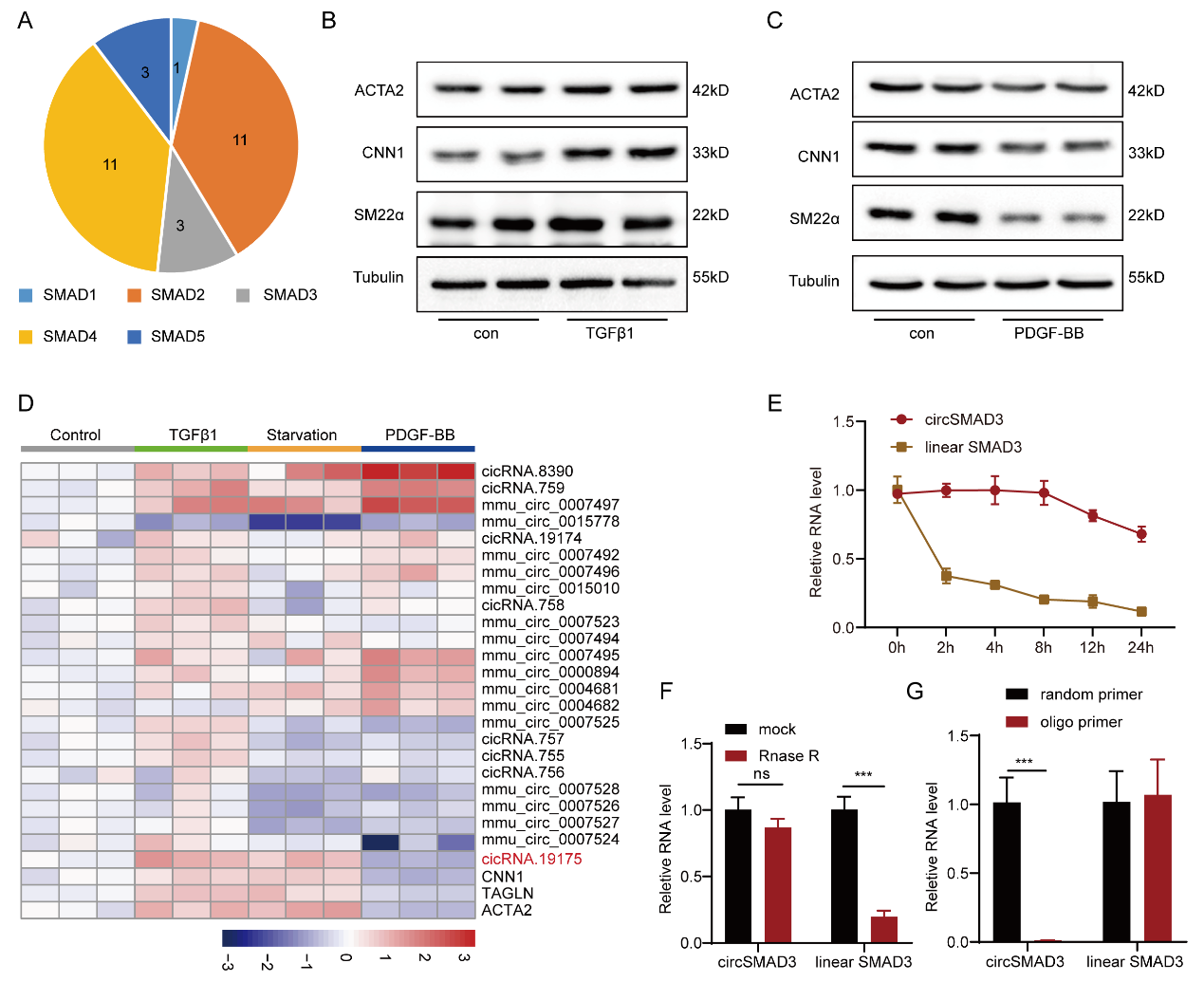


**Supplement Figure 1 Identification and properties of circSMAD3 in VSMC.** (A) circRNAs derived from SMAD family in RNA sequencing data. (B-C) The expression of contractile proteins in response to TGFβ1 (B) and PDGF-BB (C) in mASMC detected by western blot. (D) the expression of circRNAs derived from SMAD family in response to TGFβ1, starvation and PDGF-BB in mASMC detected by qPCR. (E) the changes of circSMAD3 and linear SMAD3 RNA in response to actinomycin D in HASMC. Data are shown as mean ± SD (Student's t-test). (F) the changes of circSMAD3 and linear SMAD3 RNA in response to Rnase R in HASMC. Data are shown as mean ± SD (Student's t-test). (G) qPCR assays to detect circSMAD3, mSMAD3 using random primer and oligo(dT) primer, respectively. Data are shown as mean ± SD (Student's t-test). ***p<0.001, **p<0.01, *p<0.05.


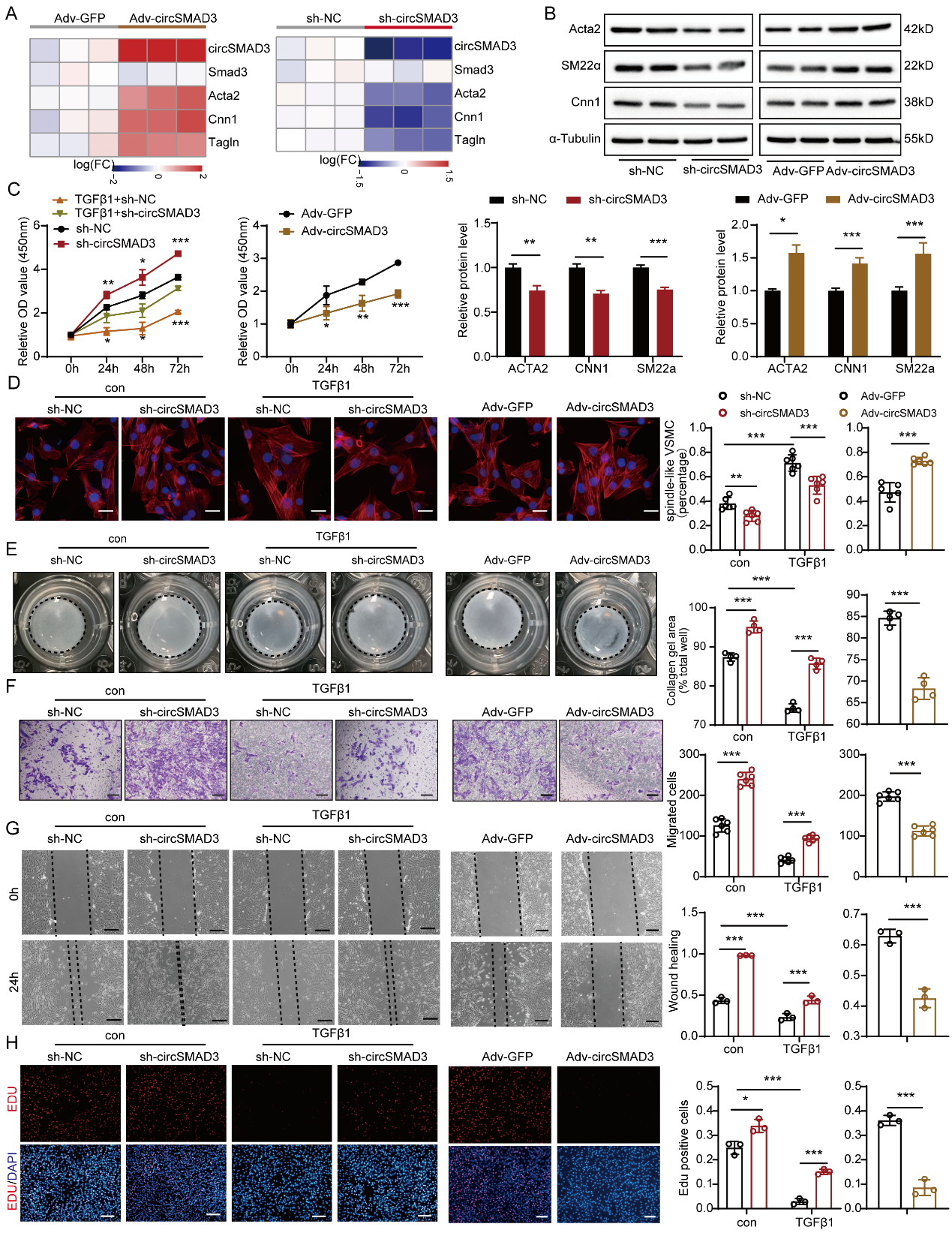


**Supplement Figure 2 circSMAD3 modulates VSMC phenotype switching and proliferation in mASMC. (A)** Heat map to show the overexpression and silence efficiency after being infected with circSMAD3 overexpression or silencing adenovirus and the expression of contractile marker genes. Data are shown as mean ± SD (Student's t-test, as indicated). **(B)** Western blot to detect contractile genes expression in protein level with circSMAD3 silence (left) and overexpression (right). Data are shown as mean ± SD (two-way ANOVA). **(C)** CCK8 assay to detect the proliferation of mASMC after circSMAD3 silence and overexpression at multiple time points (0h, 24h, 48h and 72h). Data are shown as mean ± SD (left, two-way ANOVA; right, Student's t-test). **(D)** Left, phalloidin staining to show the morphology of mASMC after circSMAD3 silence and overexpression. Scale bar=50μm. Right, the percentages of spindle-like (contractile phenotype) VSMC in each group. Data are shown as mean ± SD (left, two-way ANOVA; right, Student's t-test). **(E)** Left, Collagen gel contrition assay to evaluate the contraction of mASMC after circSMAD3 silence and overexpression. Right, the contraction rate quantified in each group. Data are shown as mean ± SD (left, two-way ANOVA; right, Student's t-test). **(F)** Left, Transwell assay to evaluate the migration of mASMC after circSMAD3 silence and overexpression. Scale bar=200μm. Right, the migration rate quantified in each group. Data are shown as mean ± SD (left, two-way ANOVA; right, Student's t-test). **(G)** Left, Wound healing assay to evaluate the migration of mASMC after circSMAD3 silence and overexpression. Scale bar=200μm. Right, the migration rate quantified in each group. Data are shown as mean ± SD (left, two-way ANOVA; right, Student's t-test). **(H)** Left, EDU assay to detect the proliferation of mASMC after circSMAD3 silence and overexpression. Scale bar=100μm. Right, the EDU positive rate quantified in each group. Data are shown as mean ± SD (left, two-way ANOVA; right, Student's t-test). ***p<0.001, **p<0.01, *p<0.05.


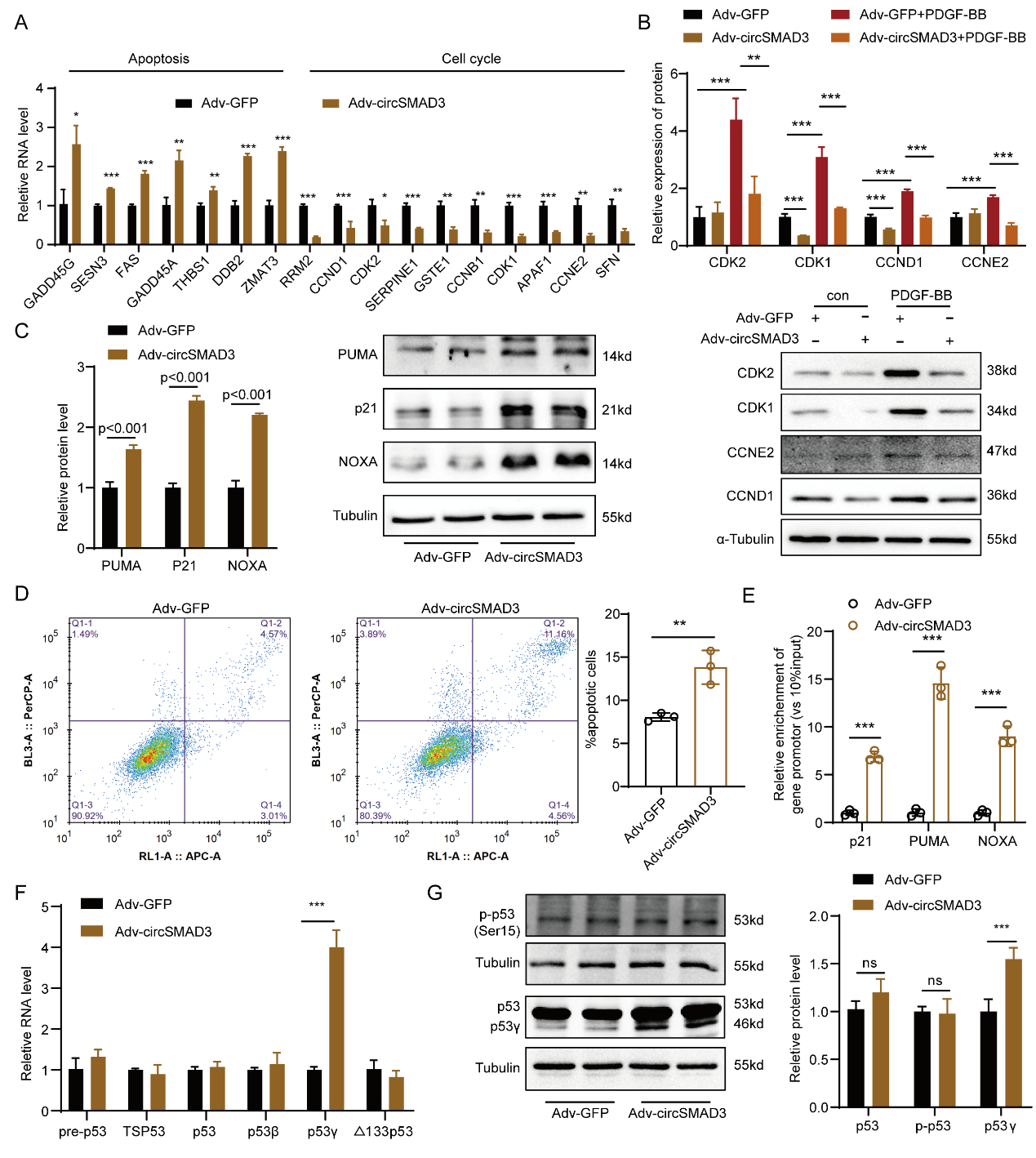


**Supplement Figure 3 circSMAD3 maintains VSMC phenotype switching through the p53γ signalling pathway**.

(A) qPCR showing the detection of the changes in apoptosis and cell cycle-related genes in the p53 signalling pathway during circSMAD3 overexpression. Data are shown as mean ± SD (Student's t-test, as indicated). (B-C) Western blot to detect proliferative gene expression (B) and pro-apoptosis related genes (C) in circSMAD3 overexpression and PDGF-BB stimulation (20 ng/mL). Data are shown as mean ± SD (two-way ANOVA, as indicated) (B) or mean ± SD (Student's t-test, as indicated) (C). (D) Flow cytometry assay to detect the apoptosis of HASMC in circSMAD3 silence. Data are shown as mean ± SD (Student's t-test, as indicated). (E) Western blot showing the detection of changes in the protein level of p53 isoform and modification level when circSMAD3 is overexpressed. Data are shown as mean ± SD (Student's t-test, as indicated). (F) RNA level of p53 isoform in response to circSMAD3 overexpression. Data are shown as mean ± SD (Student's t-test, as indicated). (G) ChIP-qPCR to detect the binding intensity of p53 on its target gene promotors when circSMAD3 overexpressed. Data are shown as mean ± SD (Student's t-test, as indicated). ***p<0.001, **p<0.01, *p<0.05.


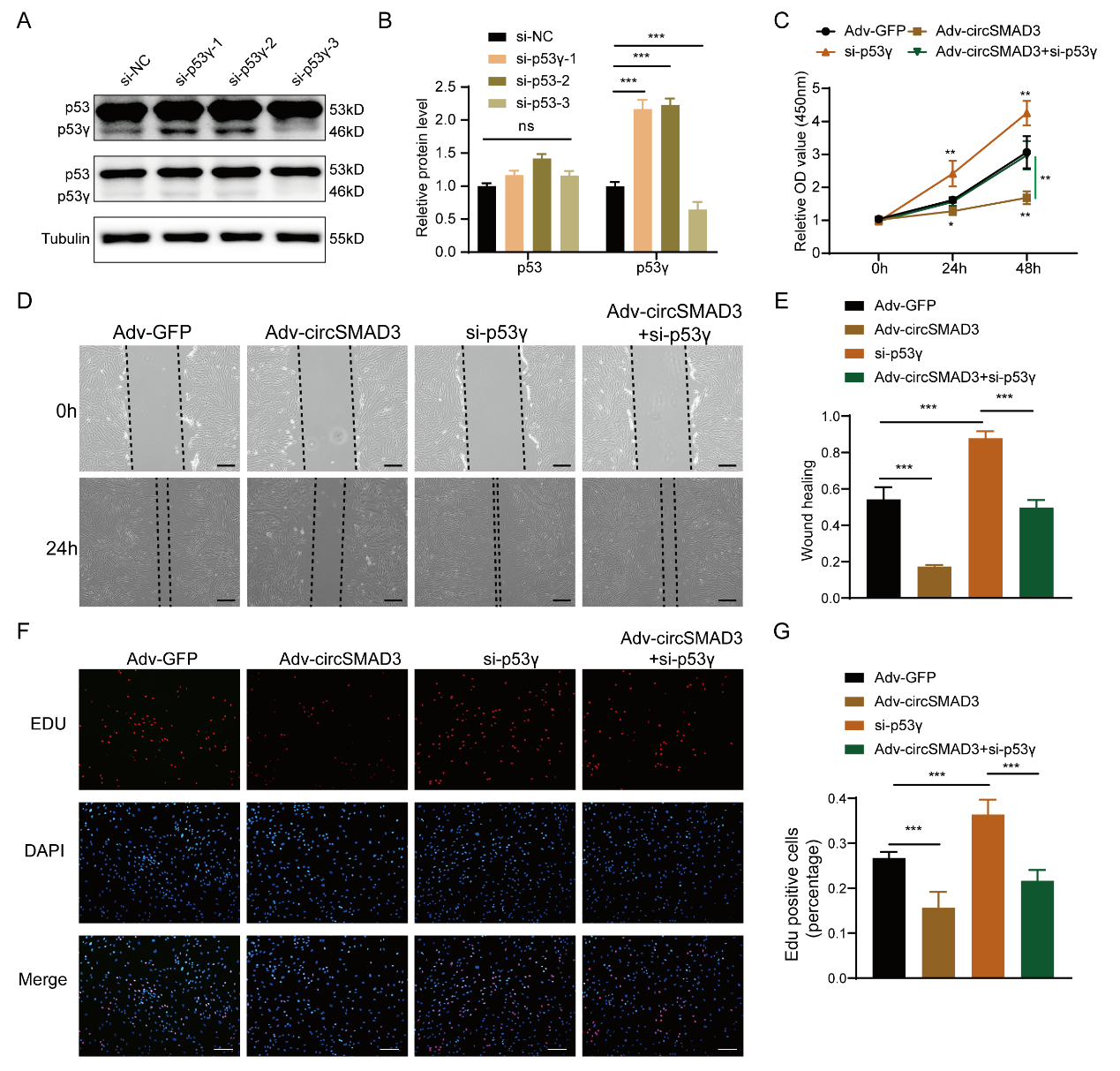


**Supplement Figure 4 circSMAD3 suppressed VSMX proliferation mediated by p53γ.** (A-B) Western blot to conformed the silence effective of p53γ siRNAs. Data are shown as mean ± SD (one-way ANOVA). (C) CCK8 to detect the function of p53γ on VSMC proliferation. Data are shown as mean ± SD (one-way ANOVA). (D-E) Wound healing assay to detect the function of p53γ on VSMC migration. Data are shown as mean ± SD (one-way ANOVA). (F-G) EDU assay to detect the function of p53γ on VSMC proliferation. Data are shown as mean ± SD (one-way ANOVA). ***p<0.001, **p<0.01, *p<0.05.
